# Delimiting priority areas for the conservation of endemic and threatened Neotropical birds using a niche-based gap analysis

**DOI:** 10.1371/journal.pone.0171838

**Published:** 2017-02-10

**Authors:** Dorinny Lisboa de Carvalho, Tiago Sousa-Neves, Pablo Vieira Cerqueira, Gustavo Gonsioroski, Sofia Marques Silva, Daniel Paiva Silva, Marcos Pérsio Dantas Santos

**Affiliations:** 1 Programa de Pós-Graduação em Zoologia, Universidade Federal do Pará / Museu Paraense Emílio Goeldi, Av. Perimetral 1901, Belém, Brazil; 2 Research Center in Biodiversity and Genetic Resources/InBIO Associate Laboratory, Campus Agrário de Vairão, Vairão, Portugal; 3 Eudocimus Consultoria Ambiental, R. 31, n° 28b, Bequimão, São Luís, Maranhão, Brazil; 4 Departamento de Biologia, Instituto Federal Goiano, Rodovia Geraldo Silva Nascimento, Urutaí, Goiás, Brazil; Centre for Cellular and Molecular Biology, INDIA

## Abstract

Knowledge of spatiotemporal distribution of biodiversity is still very incomplete in the tropics. This is one of the major problems preventing the assessment and effectiveness of conservation actions. Mega-diverse tropical regions are being exposed to fast and profound environmental changes, and the amount of resources available to describe the distribution of species is generally limited. Thus, the tropics is losing species at unprecedented rates, without a proper assessment of its biodiversity. Species distribution models (SDMs) can be used to fill such biogeographic gaps within a species’ range and, when allied with systematic conservation planning (e.g. analyses of representativeness, gap analysis), help transcend such data shortage and support practical conservation actions. Within the Neotropics, eastern Amazon and northern Cerrado present a high variety of environments and are some of the most interesting ecotonal areas within South America, but are also among the most threatened biogeographic provinces in the world. Here, we test the effectiveness of the current system of Protected Areas (PAs), in protecting 24 threatened and endemic bird species using SDMs. We found that taxa with wider distributions are potentially as protected as taxa with smaller ranges, and larger PAs were more efficient than smaller PAs, while protecting these bird species. Nonetheless, Cerrado PAs are mostly misallocated. We suggest six priority areas for conservation of Neotropical birds. Finally, we highlight the importance of indigenous lands in the conservation of Neotropical biodiversity, and recommend the development of community management plans to conserve the biological resources of the region.

## Introduction

The world is undergoing rapid and intense environmental changes that are, directly or indirectly, caused by human activities. Habitat loss and fragmentation, deposition of anthropogenic fixed nitrogenous substances, and the rise of atmospheric carbon dioxide concentration related to climatic changes are or will be the main drivers of such alterations [1,2]. Under this scenario, high-quality species distributional data are essential to set efficient conservation actions [3–5]. However, those biogeographic information are often lacking, being one of the major setbacks preventing the assessment of need and effectiveness of these actions (the Wallacean shortfall) [4,6]. Such a scenario is even more concerning in tropical regions [7–9], because these are mega-diverse areas, that have been suffering fast environmental changes [10,11], and in general, the amount of resources to describe the distribution of species is limited [12–14]. Consequently, the tropics are losing species at unprecedented rates [15–17], often without properly identifying and describing their biodiversity (the Linnean shortfall) [4,18].

One way to fight back against the Wallacean shortfall is to use species distribution models (SDMs) [19–21]. These models correlate known occurrences of target species with climatic, land-use, and topographic data to delimit the multidimensional bioclimatic requirements for the modeled taxa, reflecting their environmental preferences [22]. SDMs can be overlaid upon the geographic range space to fill biogeographic gaps within species’ ranges [23], even for elusive and seldom recorded species [24–27]. In conservation, SDMs have been widely and successfully used to 1) predict the distribution of rare, endemic and threatened species [25,28–31], 2) perform niche-based gap analyses and discover species that are not protected (i.e. do not occur in any protected area; PA hereon) [32–34], 3) predict suitable areas for the invasion of exotic species [35–38], 4) evaluate the potential effects of future climate changes [39,40], 5) determine suitable areas for the reintroduction of rescued fauna [41,42], and 6) establish and evaluate priority areas for conservation [43–45], amongst other examples.

Therefore, one of the first steps for setting a conservation plan may be to ally SDMs with systematic conservation planning [46], particularly in the analysis of representativeness, also known as gap analysis [20,34,47–49]. Gap analysis consists of the identification, classification, and examination of the existing system of PAs based on the assessment of the representation of species, vegetation types or biomes within those PAs network and identification of gaps of distribution in its coverage [23,50]. Representativeness is one of the four main principles of systematic conservation planning, the others: comprehensiveness, adequacy and efficiency [46,51–55].

**Fig 1:**
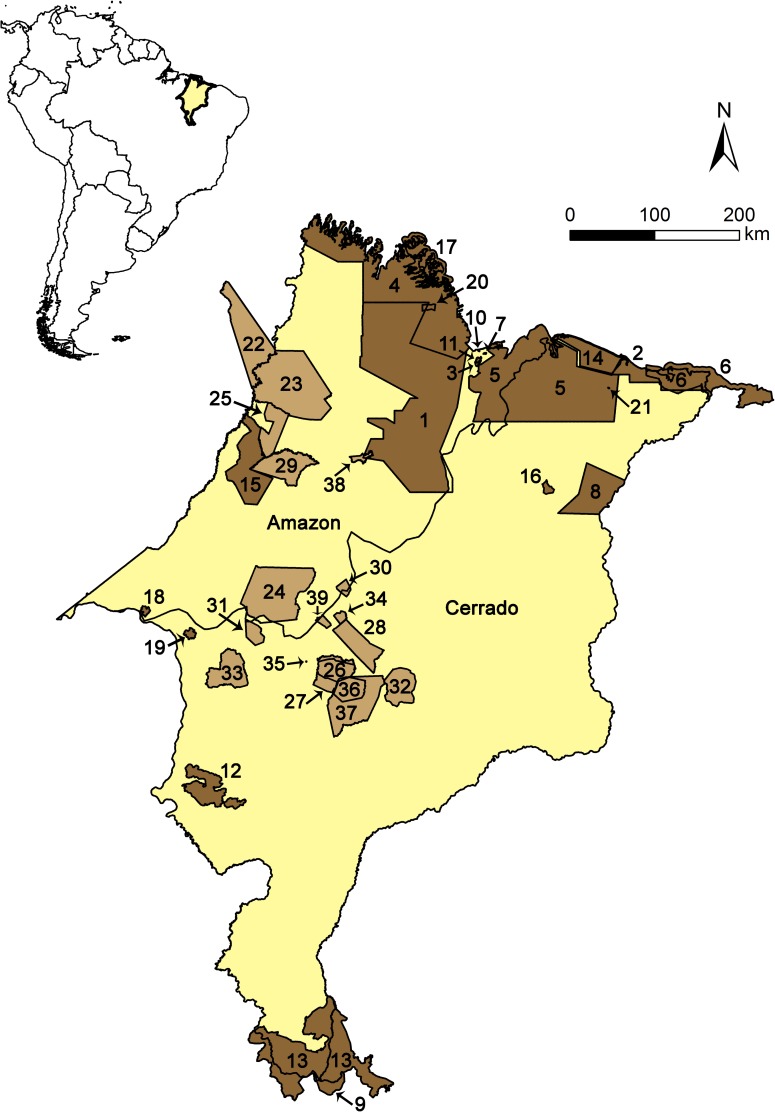
Map of the study area. Location of Protected Areas (PAs): conservation units (dark brown) and indigenous lands (light brown), within the Amazon and Cerrado biomes. See Table 1 for PAs identification (ID).

The region encompassing eastern Amazon and northern Cerrado (Fig 1) is one of the most heterogeneous regions throughout the Amazon basin. This area presents a high variety of environments, as tropical rainforests (*terra firme* and *várzea*), floodplains, campinas, extensive mangroves in the coastal zone, being a large ecotonal area with the Cerrado [56–58]. All this diversity has been affected by intense anthropogenic pressure, mainly due to high deforestation rates and a strong expansion of agribusiness. Noteworthy, 61% of the endangered birds in the Brazilian Amazon occur primarily or exclusively in this portion of the Amazon basin, which constitutes the Belém area of endemism (BAE) [59], while Cerrado is the second most threatened biodiversity hotspot of Brazil [3,60–63].

Within Eastern Amazon, the state of Maranhão (Fig 1) has already lost about 30% (24,412 km^2^) of its historical Amazon forest, and 21% (436 km^2^) of Cerrado's native vegetation [64]. Nonetheless, this is the less affected area (within BAE and Cerrado), and still preserves one of the richest avifauna of Brazil, mainly due to the aforementioned diversity of ecosystems [65,66]. The birds’ list of Maranhão has over 640 species [67], corresponding to 34% of the total number of species in Brazil [68]. Of those, 49 (21%) are included on the Brazilian official list of threatened species [59], and several taxa from the BAE have the highest level of local vulnerability [67]. The state further includes 10 endemic bird species within Cerrado (DL Carvalho et al. in prep). These features make Maranhão an interesting region to test the effectiveness of the state system of PAs (both in Amazonian forest and the Cerrado biomes), in protecting threatened and endemic bird species using SDMs. Specifically, here we used SDMs to perform a gap analysis, and seek to know if 1) taxa with relatively wider distributions are more protected (i.e. have higher percentage of area within PAs) than taxa with smaller distributions and 2) if relatively larger PAs are more efficient (i.e. have higher species richness) than smaller PAs. Finally, based on our results, we also suggested new conservation areas for the target taxa and discuss the effective implementation of new conservation practices in the Neotropical region, in order to allow a more significant conservation of its bird species.

## Materials and methods

### Study area

The study area has a total area of 331,983.29 km^2^, corresponding to the Brazilian state of Maranhão, the eighth largest Brazilian state. Political borders are biologically meaningless, but conservation actions mostly depend on political actions, so we chose to use this political delimitation to study a biologically relevant ecotonal area, located between the Amazon forest (west), Cerrado (south and southwest), and small patches of Caatinga biome (east) (Fig 1) [69]. The state’s economy is structured in two main areas of development and integration: extensive cattle ranching and logging in the Amazonian portion; and mineral and metallurgical complex, agriculture and production of energy in the Cerrado [70]. As aforementioned, despite the intense anthropogenic actions, Maranhão holds one of the largest patches of forest within BAE, and native Cerrado vegetation [64].

In this study, we considered 39 protected areas (PAs) distributed along the biomes of Amazon and Cerrado (Fig 1). Of these, 24 are conservation units: 13 are Federal PAs (eight of sustainable use and four of full protection), while 10 are state PAs (six of sustainable use and two of full protection). The other 18 are indigenous lands delimited and homologated by the Federal government (Table 1).

**Table 1:** Protected areas in the study area. Instance, kind of usage, biome, extent area according to World Wildlife (www.worldwildlife.org), and priority according to MMA [71].

_ID_	_Protected areas_	_Instance_		_Usage_		_Biome_		_Area (km_^2^_)_	_Priority_
		_Federal_	_State_	_Sustainable use_	_Full protection_	_Amazon_	_Cerrado_		
_1_	_APA Baixada Maranhense_		_X_	_X_		_X_	_X_	_17285_	_EH_
_2_	_APA Foz Do Rio Das Preguiças and, Pequenos Lençóis Região Lagunar Adjacente_		_X_	_X_			_X_	_2062_	
_3_	_APA Região Do Maracanã_		_X_	_X_		_X_		_22_	
_4_	_APA Reentrâncias Maranhenses_		_X_	_X_		_X_		_26285_	_EH_
_5_	_APA Upaon-Açú, Miritiba and, Alto Preguiças_		_X_	_X_		_X_	_X_	_14567_	
_6_	_APA Delta Do Parnaíba_	_X_		_X_			_X_	_3076_	
_7_	_APA Itapiracó_		_X_	_X_		_X_		_4_	
_8_	_APA Morros Garapenses_		_X_	_X_			_X_	_2343_	
_9_	_APA Serra Da Tabatinga_	_X_		_X_			_X_	_352_	
_10_	_ESEC Sítio Rangedor_		_X_		_X_	_X_		_1_	
_11_	_Estadual Park Bacanga_		_X_		_X_	_X_		_26_	
_12_	_PARNA Chapada das Mesas_	_X_			_X_		_X_	_1600_	
_13_	_PARNA Nascentes do Rio Parnaíba_	_X_			_X_		_X_	_7243_	
_14_	_PARNA Lençóis Maranhenses_	_X_			_X_	_X_	_X_	_1566_	
_15_	_REBIO Gurupi_	_X_			_X_	_X_		_2903_	_EH_
_16_	_RESEX Chapada Limpa_	_X_		_X_			_X_	_120_	
_17_	_RESEX Cururupu_	_X_		_X_		_X_		_1852_	
_18_	_RESEX Ciriaco_	_X_		_X_		_X_		_81_	
_19_	_RESEX Mata Grande_	_X_		_X_			_X_	_114_	
_20_	_RESEX Quilombo Frechal_	_X_		_X_		_X_		_93_	_EH_
_21_	_RPPN Prata_^a^						_X_	_1_	
_22_	_Alto Rio Guamá_					_X_	_X_	_2799_	
_23_	_Alto Turiaçu_						_X_	_5305_	_EH_
_24_	_Araribóia_					_X_		_4133_	_EH_
_25_	_Awa_						_X_	_1166_	_EH_
_26_	_Bacurizinho_^b^						_X_	_134_	_EH_
_27_	_Bacurizinho_						_X_	_840_	_EH_
_28_	_Cana Brava and, Guajajara_					_X_	_X_	_1373_	_EH_
_29_	_Caru_					_X_	_X_	_1727_	_EH_
_30_	_Geralda Toco Preto_						_X_	_185_	_EH_
_31_	_Governador_						_X_	_416_	_EH_
_32_	_Kanela_					_X_		_1252_	
_33_	_Krikati_					_X_		_1448_	
_34_	_Lagoa Comprida_					_X_	_X_	_132_	_EH_
_35_	_Morro Branco_						_X_	_48_	
_36_	_Porquinhos_^b^						_X_	_795_	
_37_	_Porquinhos dos Canela and, Apanjekra_					_X_		_3010_	
_38_	_Rio Pindaré_					_X_		_150_	
_39_	_Urucu / Juruá_	_ _	_ _	_ _	_ _	_X_	_X_	_127_	_EH_

^a^Private area created by voluntary act of owner and established by government.

^b^Overlaid area composed by different tribes.

APA, Environmental Protection Area; ESEC, Ecological Station; PARNA, National Park; REBIO, Biological Reserve; RESEX, Extractive Reserve; RPPN, Private Reserve of Nature Patrimony. EH, Extremely high.

### Target species and occurrence dataset

Our total dataset includes 24 terrestrial, non-migratory bird taxa, with enough occurrence records available (Table 2). Fourteen are classified as threatened in the Belém area of endemism (BAE) in Amazonia [59,72], and 10 are endemics to the Cerrado biome [73–76], including two species considered Vulnerable to extinction [59,72].

**Table 2:** Modeled bird taxa. Points: number of records (4.5 × 4.5 km cells), AUC and TSS: mean values, and respective standard deviation values, ER/study area: estimated range in number of cells in the study area, ER/biome: estimated range in number of cells in the study area by biome (Amazon/Cerrado), %PA/biome: percentage of occurrence in protected areas, Status: conservation status according to IBAMA[59] and IUCN[72], and biome of occurrence.

Taxon	English name	Points	AUC	TSS	ER/study area	ER/biome	% PA/biome	Status^b^	Biome
*Crax f*. *pinima*^a^	Bare-faced Curassow	5	0.97±0.00	0.96±0.01	498	496	52	CR	Amazon
*Psophia obscura*^a^	Dark-winged Trumpeter	7	0.97±0.00	0.87±0.21	1420	1420	63	CR	Amazon
*Guaruba guarouba*	Golden Parakeet	20	0.96±0.01	0.80±0.18	2976	2168	60	VU	Amazon
*Pyrrhura coerulescens*	Pearly Parakeet	31	0.92±0.03	0.65±0.14	3488	3385	42	VU	Amazon
*Neomorphus geoffroyi*^a^	Rufous-vented Ground-Cuckoo	8	0.95±0.01	0.89±0.02	5270	3641/1801	51/43	VU	Amazon/Cerrado
*Pteroglossus b*. *bitorquatus*	Red-necked Aracari	30	0.95±0.02	0.77±0.12	2581	2470	54	VU (EN)	Amazon
*Celeus obrieni*	Kaempfer's Woodpecker	37	0.93±0.02	0.75±0.09	369	369/3450	93/10	VU (EN)	Amazon/Cerrado
*Piculus paraensis*^a^	Belem Golden-green Woodcreeper	9	0.97±0.00	0.93±0.01	2454	2082	60	EN (LC)	Amazon
*Phlegopsis n*. *paraensis*	Black-spotted Bare-eye	35	0.97±0.02	0.83±0.10	1374	1402	57	VU^c^	Amazon
*Hylopezus paraensis*	Snethlage's Antpitta	23	0.95±0.01	0.83±0.10	3078	2317	54	VU (LC)	Amazon
*Dendrocincla m*. *badia*	White-chinned Woodcreeper	18	0.97±0.01	0.90±04	250	246	74	VU^c^	Amazon
*Dendrexetastes r*. *paraensis*^a^	Cinnamon-throated Woodcreeper	9	0.98±0.00	0.96±00	827	827	70	EN^c^	Amazon
*Dendrocolaptes medius*	Todd's Woodcreeper	46	0.93±0.02	0.74±0.06	6157	4105	43	VU (LC)	Amazon
*Hylophilus o*. *rubrifrons*	Tawny-crowned Greenlet	34	0.79±0.02	0.77±0.08	1197	1235	58	-^c^	Amazon
*Alipiopsitta xanthops*	Yellow-faced Parrot	70	0.83±0.03	0.51±0.10	4	4	0	(NT)	Cerrado
*Cercomacra ferdinandi*	Bananal Antbird	31	0.95±0.02	0.75±0.09	1430	981	3	VU	Cerrado
*Herpsilochmus longirostris*	Large-billed Antwren	67	0.85±0.02	0.55±0.07	52	49	4	(NT)	Cerrado
*Melanopareia torquata*	Collared Crescentchest	46	0.79±0.05	0.47±0.09	4768	4102	12	(NT)	Cerrado
*Antilophia galeata*	Helmeted Manakin	70	0.84±0.03	0.53±0.10	360	375	15	(LC)	Cerrado
*Suiriri affinis*	Chapada Flycatcher	27	0.85±0.02	0.61±0.05	1362	799	18	(LC)	Cerrado
*Cyanocorax cristatellus*	Curl-crested Jay	97	0.93±0.04	0.47±0.06	5403	5214	11	(LC)	Cerrado
*Charitospiza eucosma*	Coal-crested Finch	73	0.80±0.05	0.51±0.06	8078	7360	11	VU	Cerrado
*Saltatricula atricollis*	Black-throated Saltator	110	0.82±0.03	0.44±0.21	8209	6561	10	(LC)	Cerrado
*Porphyrospiza caerulescens*	Blue Finch	26	0.78±0.06	0.55±0.16	2766	2399	6	(NT)	Cerrado

^a^Jackniffe approach result p<0.05.

^b^IUCN status is in parentheses when different from IBAMA's.

^c^No IUCN status.

Abbreviations: IUCN, International Union for Conservation of Nature; IBAMA, Instituto Brasileiro do Meio Ambiente e dos Recursos Naturais Renováveis; CR, Critically Endangered; EN, Endangered; VU, Vulnerable; NT, Near Threatened; LC, Least Concern.

We gathered distribution data for each taxon from literature records, online databases [VertNet (http://vertnet.org/), Species Link (http://splink.cria.org.br), Global Biodiversity Information Facility (http://www.gbif.org), Wikiaves (http://www.wikiaves.com.br), xenocanto (http://www.xeno-canto.org)], museum collections (Louisiana Museum of Natural History, Museu Paraense Emílio Goeldi and Museu Nacional do Rio de Janeiro) and personal observation (DLC, GG, and PVC; see S1 Fig for a complete list of records). We checked all occurrences and excluded dubious records based on the known distribution of the species [72]. Geographical coordinates were obtained directly from the original sources or from Ornithological Gazetteer of Brazil [77]. Bird nomenclature follows the Brazilian Ornithological Records Committee [68].

### Environmental layers, modeling procedures, thresholds and evaluation

Occurrence records were overlaid on grid of cells of 2.5 arc-min (~4.5 x 4.5 km). A buffer of 200 km was set around all records to define total extent area (S2 Fig). Using this same grid and considering all 19 bioclimatic variables from WorldClim (http://www.worldclim.org/), we performed a pair-wise Pearson correlation test of all variables to remove those highly correlated and reduce their collinearity [78]. In the case of high correlation (r> 0.8 or r< -0.8), we used only one of the variables in the distribution modeling. We selected nine predictor variables as our environmental variables (Annual Mean Temperature, Mean Diurnal Range, Isothermality, Max Temperature of Warmest Month, Annual Precipitation, Precipitation of Driest Month, Precipitation Seasonality, Precipitation of Warmest Quarter and Precipitation of Coldest Quarter).

All models were trained with MaxEnt 3.3.3 [79,80]. This method computes the suitable distribution of maximum entropy for the set of climatic variables associated to the occurrence records of the target species, however this procedure can be constrained by the incomplete knowledge about the distribution of the species [79,81]. MaxEnt is a presence/background method that requires only presence data as input, and consistently performed well in comparison to other methods [48], especially at low samples sizes [28,82,83].

Due to limited availability of suitable occurrence data for five modeled taxa (<10 records), we used the Jackknife approach, also known as leave-one-out method [28], to predict their potential distributions. Then, we evaluated the resulting distributions with the same subsets. For the remaining 19 taxa, we used 10 subsets dividing the occurrences into 70% training and 30% testing records. We used the threshold that balances both omission and commission errors while modeling the species distributions to cut the suitability matrices of the modeled species in modeling algorithm into presence-absence maps [84,85]. The statistically significant probability (*p*<0.05) indicate that the model predictions are reliable, despite some eventual omission and/or commission. All probabilities were evaluated in R 3.3.1 (www.r-project.org). We further used both Area Under the receiver–operator Curve (AUC) [86] and the True Skilled Statistics (TSS) [87] to assess models’ performance. AUC and TSS account for the sensitivity (quantifies omission errors), and specificity (quantifies commission errors) of the models [87]. AUC values vary between 0 and 1, with values ≤0.5 representing models no better than random and values around 1 representing a perfect fitting between the observed and the predicted species distribution. Thus, we considered acceptable distribution models to be those with AUC≥0.7 [86,88]. TSS varies from −1 to +1, where negative and around zero values indicate that distributions are no better than random, while values near +1 represent perfect agreement between the observed and the modeled distributions. Acceptable and excellent models were those with TSS values of at least 0.5 and ≥0.7, respectively [87]. We used 10.000 random pseudo-absences in all model evaluation procedures. A mean consensual distribution map for each taxon was made with those models which achieved TSS >0.4. This method was considered to be the best to delimit the final distribution of a given taxon for several different modeling algorithms [89].

### Estimated protected range, species richness and identification of priority areas for conservation

As our group of target taxa occurs in two distinct biomes, one predominantly forested (Amazon) and another dominated by savanna (Cerrado), it is expected that predominantly forest-dependent taxa from the Amazon biome are not present in protected areas dominated by savanna, while endemic species from Cerrado are not expected to occur in PAs dominated by forest. Accordingly, we considered two different approaches to estimate the proportion of protected area for each taxon, and species richness, and so to perform the statistical tests related to the gap analysis. In the first approach, we considered all 24 taxa together, and in the second, taxa from each biome were considered separately. *Neomorphus geoffroyi* and *Celeus obrieni* were considered in both Amazon and Cerrado analyses, since each are known to occur in both biomes. We obtained the modeled species richness by summing the final distribution of each taxon.

We used the shapefile of the world ecoregions available at the World Wildlife website (http://www.worldwildlife.org/publications/terrestrial-ecoregions-of-the-world) to depict PAs (conservation units and indigenous lands) within the study area. PAs were converted to raster files with grid cells of approximately the same resolution used in the modelling procedures (0.041° or ~4 km near the Ecuador). We identified the grid cells in which each bird taxon was predicted to occur in protected area.

We used linear regressions and power functions to evaluate the effectiveness of PAs. To evaluate the relationship between the size of PAs individually and the estimated species richness covered in each PAs, we used the same two approaches, and so considered three scenarios: 1) the maximum value of Amazon species richness present in the Amazon biome; 2) the maximum value of endemic Cerrado's species richness present in Cerrado; 3) the maximum value of all target taxa richness throughout the study area. A 95% confidence interval for the slope for all regression analyses was selected.

Finally, to identify priority areas for conservation, we overlaid the predicted species richness for each biome separately with maps of deforestation in the Amazon biome (PRODES data from [64]), and remnants of native vegetation in Cerrado [90]. Protected Areas fully covered by native vegetation, and in which more than half of the target taxa potentially occurs, were considered priority areas for conservation.

## Results

### Species distribution models

**Fig 2:**
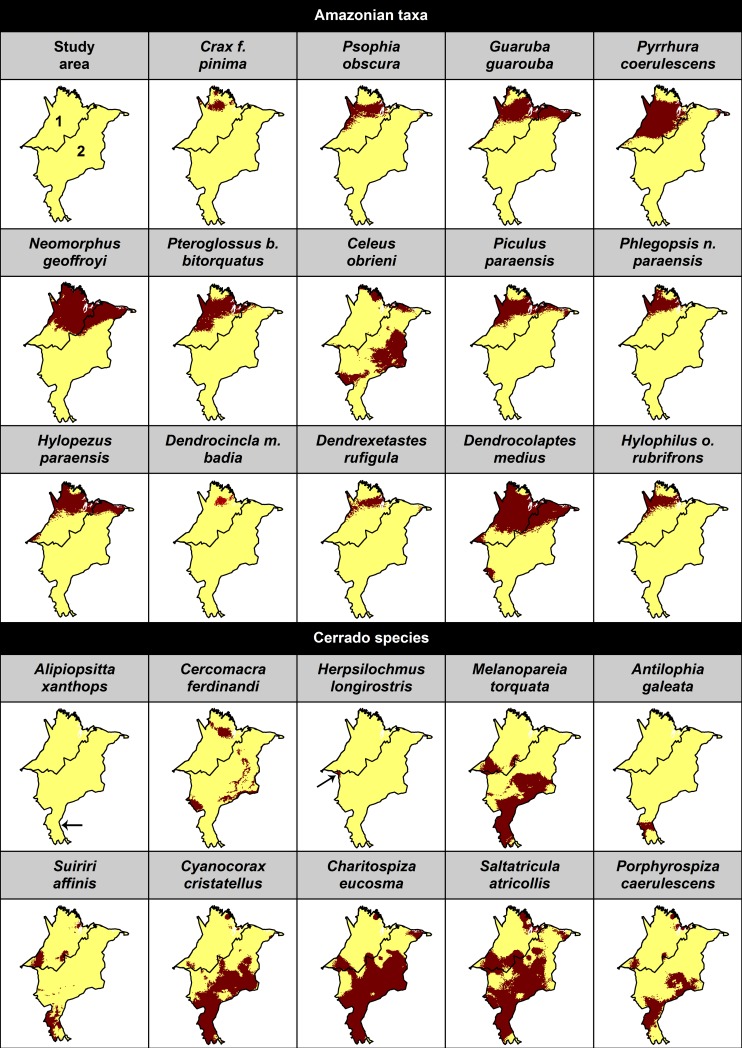
Species distribution models for the study area. Estimated range of taxa from Amazon 1) and Cerrado 2) biomes.

We collected a total of 1,534 occurrence records, from which 929 were used (sample size varied between 5 and 110; S1 Fig) to generate the potential distribution maps of the 24 target bird taxa (S2 Fig; Fig 2). Six out of the 24 SDMs presented errors of omission and/or commission (*Dendrocincla m*. *badia*, *Alipiopsitta xanthops*, *Herpsilochmus longirostris*, *Antilophia galeata*, *Suiriri affinis* and *N*. *geoffroyi*). SDMs for *A*. *xanthops*, and *H*. *longirostris* had considerably reduced potential area of occurrence within the study area (Fig 2; S2 Fig). Yet, all SDMs showed fair to excellent predictive capability (Table 2). AUC values varied between 0.78 and 0.98. TSS values were always higher than 0.5, except for the endemic species from Cerrado, *Saltatricula atricollis* (TSS = 0.4). Models for taxa with less than 10 records (*Crax f*. *pinima*, *Psophia obscura*, *Neomorphus geoffroyi*, *Piculus paraensis*, *Dendrexetastes r*. *paraensis*) predicted taxa distributions better than random (*p*<0.05), according to the leave-one-out method (Table 2). Thus, all taxa were considered in the following analyses. In the study area, predominantly forest-dependent taxa had higher probabilities of occurrence in the Amazon biome, and Cerrado endemics were mostly assigned to occur in this biome (Fig 2). Also, as expected, *C*. *obrieni* was predicted to be present in both biomes. However, SDM for *N*. *geoffroyi* estimated the distribution of this species to be mostly restricted to the Amazon (Fig 2). Therefore, further results were mostly focused in the approach separating taxa by biome (with *N*. *geoffroyi* excluded from analyses considering Cerrado taxa), and results considering total target taxa and the entire study area are only presented for comparison.

### Estimated protected range and species richness

Percentage of protected area for each taxon varied between 41% (*Pyrrhura coerulescens*) and 94% (*C*. *obrieni*) considering the Amazon biome only, and 0% (*A*. *xanthops*) and 11% (*S*. *affinis*) for Cerrado taxa (Table 2).

**Fig 3:**
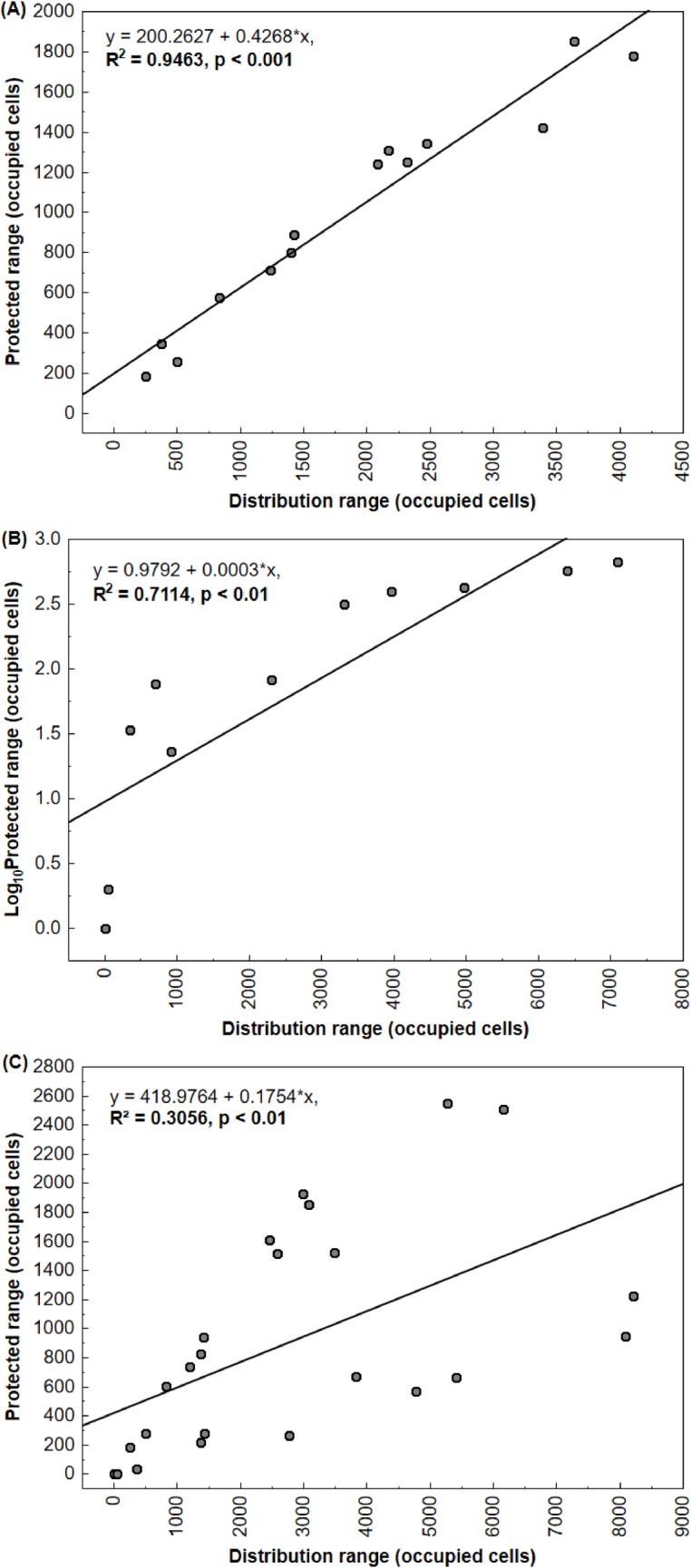
Distribution of protected range in relationship to total range size within the study area. We observed a positive relationship between the total range and the protected range size of threatened taxa from Amazon (A), Cerrado (B) and all target species in the whole study area (C).

Our linear regression analyses indicated that both conservation units and indigenous lands are protecting the target taxa better than random (Fig 3). For the species within the Amazon, for every 202.5 km^2^ of distribution range (10 grid cells), there was a gain of protection of about 81 km^2^ (four cells; Fig 3A). On average, 59%±13% of estimated ranges for the Amazonian species is protected in this biome. In Cerrado portion of the study area, for every ≈69.000 km^2^ (3400 cells of distribution), there was a gain of protection of only 20.25 km^2^ (one cell; Fig 3B). The protected range of species from Cerrado averaged only 12%±11% of their distribution. Using the entire study area and the total dataset of target taxa, we obtained a gain of protection of 40.5 km^2^ (two cells), for every 202.5 km^2^ (10 cells of distribution) (Fig 3C). For all 24 species in the whole study area, the average of protected range was 38%±26%.

**Fig 4:**
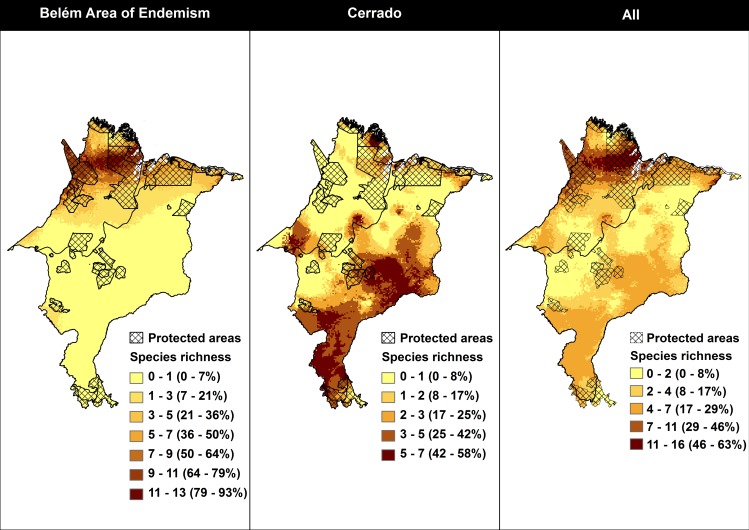
Estimated species richness in relationship to protected areas in study area.

**Fig 5:**
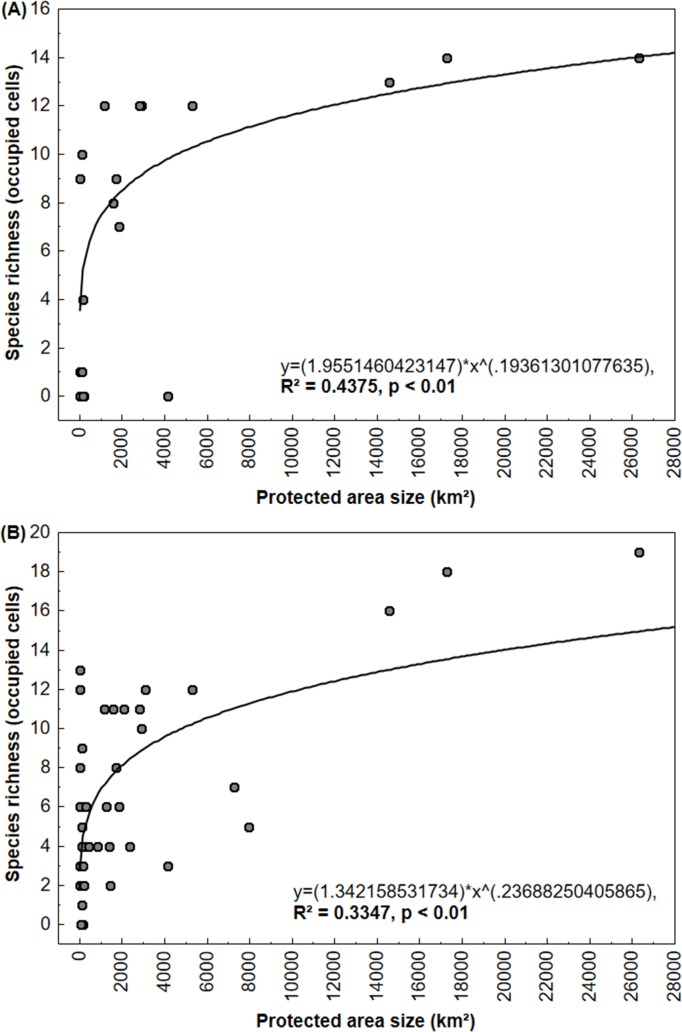
Distribution of species richness in relationship to the size of each protected area individually. A) Amazon biome and B) the whole study area.

Considering Amazonian threatened taxa (n = 14), within this biome, we observed that areas with higher estimated species richness (n≥7, i.e. ≥50%) are located in the north-western region of the study area (Fig 4A). The relationship between the sizes of each PA and the estimated species richness in this biome was positive (Fig 5A), i.e. the largest protected areas in this region have a wider number of species. For each 2.025 Km^2^ (100 grid cells), a gain of protection of one species was obtained (Fig 5A). Regarding only the Cerrado’s potential species richness, areas with higher values (n≥6, i.e. ≥50%) are inserted in patches in the southeast and south of the study area (Fig 4B). There was no relationship between the size of PAs of this biome and the estimated species richness, since the random model was sufficient to explain the observed variation (*R*^*2*^ = 0.201, *p >*0.05, *y =* 0.8253*x^0.2276).

Considering the 24 target taxa together in the entire study area, the highest values of species richness (n≥12, i.e. ≥50%) were in the Amazon biome, and only some patches were highlighted in Cerrado, mainly in the southern part of the study area (Fig 4C). Within Cerrado, the estimated species richness with all target taxa reached a maximum of only 16% (Fig 4C). The relationship between estimated species richness and the size of each PA in the entire study area was positive (Fig 5B). For each 1.012 Km^2^ (50 grid cells) a gain of protection of one taxon was obtained (Fig 5B).

### Identification priority areas for conservation

**Fig 6:**
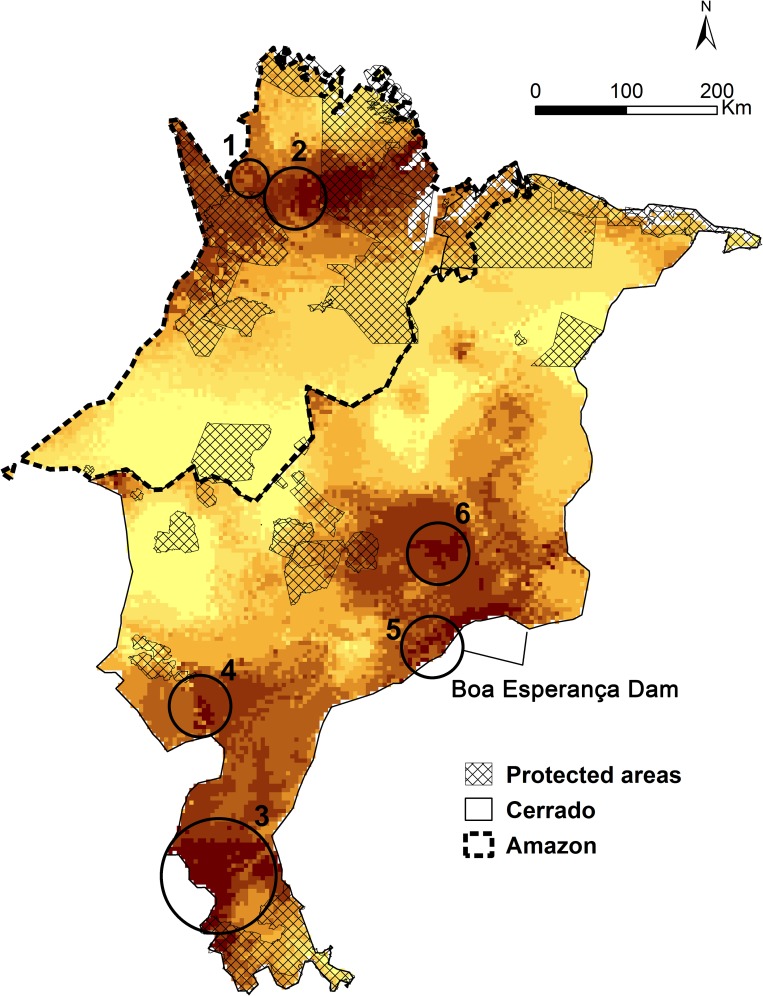
Priority areas for conservation. Study area showing the currently existing protected areas and indicating new priority areas for conservation according to the estimated species richness in the Amazon and Cerrado biomes, separately. 1—Regions connecting APA Baixada Maranhense with indigenous land Alto Turiaçu; 2 –Extension of indigenous land Alto Turiaçu; 3—Polígono das Águas in southern Maranhão; 4—Southwestern Plateau; 5—Mirador/ Uruçuí; 6—Extension of the Maranhão semideciduous forest area, in central Maranhão.

Again, using the approach of analyzing Amazon and Cerrado, and their taxa, separately, we highlight two priority areas for conservation in the Amazon biome, and four areas in Cerrado (Fig 6). As aforementioned, these priority areas for conservation have a species richness ≥50% and still maintain native vegetation.

## Discussion

### Species distribution models

Overall, our species distribution models (SDMs) agree with known distributions and ecological requirements of target taxa. Most target Amazon taxa have a high specificity for forested habitats [67], and in fact had predicted distributions mostly restricted to those regions. Only for *Crax f*. *pinima*, *Guaruba guarouba*, *Neomorphus geoffroyi*, *Hylopezus paraensis* and *Dendrocolaptes medius*, SDMs predicted their occurrence also in Cerrado, although these are forest-dependent taxa. In fact, these were predicted to occur in mangrove areas in coastal zones, because of the similar climatic features of mangroves and the neighboring rainforests. Cerrado and open areas taxa had SDMs consistent with their known habitat affinity [91–93]. For conservation purposes, models that estimate the full niche requirements are preferred [34]. Thus, our SDMs express the full niche of our target taxa, and so are adequate to perform gap analysis [86,87], even for taxa with low numbers of records (this study; [28,94]). As detailed in the next sections, we add support to the use of SDMs in the systematic conservation planning of Neotropical organisms, as previously shown not only for birds, but also other different taxonomic groups such as odonata [34], anura [6], turtles [49], and mammals [95].

Nonetheless, we know that the potential distribution maps are an abstraction that might not reflect species occurrence at fine geographic scales [96,97]. Furthermore, in ecotonal areas such as ours, which might represent a limit of distribution for several distinct taxa, species might have different ecological requirements than at the core of their distributions, and do not fully express their niches (incomplete niche expression), and finer-scale studies may be necessary to addresses some species-specific questions [98,99]. This is particularly important in the current fast climatic changing scenario all natural species are facing. As our SDMs show, Amazonian and Cerrado birds seem to have totally distinct climatic requirements, and so may respond differently to climate change. Thus, to decrease the Wallacean shortfall, we propose that more studies are necessary in such transition regions in the Neotropics, considering the lack of information and that the ecotonal condition may promote a high species richness [100].

### Species estimated protected distribution

Taxa with broad distributions are potentially as protected as taxa with smaller distributions within the study area. Nonetheless, despite the positive relationship between the amount of potential range and the amount of potential range that is protected, this latter proportion varied enormously between biomes.

Taxa with a potential area of distribution wider than 250,000 km^2^ must have at least 10% of its distribution protected, and taxa with smaller distributions (around 10^3^ km^2^), should be fully protected, i.e. 100% of its potential distribution must be included in PAs [101]. Within the study area, most target taxa had potential distributions greater than 10^4^ km^2^. Thus, no less than about 60% of their potential ranges should be protected [102]. For the target Amazonian taxa, this is close to the mean percentage of potential protected area estimated, suggesting these species are well protected. However, the Critically Endangered *C*. *f*. *pinima* and *P*. *obscurus*, and the Threatened *P*. *paraensis*, *N*. *geoffroyi* and *D*. *r*. *paraensis* occur at low population densities, even in well-preserved areas, and most of them have already been indicated as likely extinct at a regional level, even in still forested areas, due to degradation and hunting, particularly in western BAE [67,103–108]. This implies that, despite our results on linear regression analyses, these taxa might need further conservation actions. More importantly, they exemplify the need to gather the most up to date information available on Neotropical species; otherwise more recent impacts of habitat loss and degradation might be overlooked.

Within the Cerrado portion of the study area, the mean potential area of distribution estimated to be currently protected was only of 12%; a percentage close to the 10% recommended by Rodrigues’ et al.[101] for widespread species. Yet, it is alarming that, even considering the whole biome, hardly any Cerrado species will overcome this threshold, because current PAs system within Cerrado is highly inefficient in conserving bird species [39]. Only 12 (32%) of the target species analyzed by Nóbrega & De Marco [33] had 5% of their distribution protected, even when authors considered all Brazil, and none of them even reached the 10% threshold if considering only large reserves [33]. Furthermore, protected potential distribution of Cerrado species might be insufficient to maintain viable populations, due to the high level of fragmentation, especially within the southern part of the biome [109]. In fact, endemic Cerrado birds are already presenting signs of a decreasing gene flow due to anthropogenic habitat fragmentation and degradation [110]. Moreover, Cerrado endemics have highly specific habitat requirements, such as the Vulnerable *C*. *ferdinandi* [59,72,111]. Its potential protected distribution was estimated to be only 3% in the study area, despite occurring in more than about 22,000 km^2^. The demand for a specific conservation plan for this species was already stressed elsewhere [39], and our results further support this recommendation.

Our study reinforces the need to overcome the huge Wallacean shortfalls that prevents proper conservation planning of Neotropical species. Assessments based on species-specific information, not only occurrence data, but also biological and ecological data, should be added to general conservation plans [46], and must be thorough and updated frequently, due to fast land-use changes. Not only the Amazon and Cerrado are losing native vegetation at a fast rate, but many other Neotropical regions are equally or more threatened. For instance, the Brazilian Atlantic forest, the tropical Andes, and the Chilean Winter Rainfall-Valdivian Forests were, almost two decades ago, highlighted as hotspots of biodiversity [3], and their degree of threat has still not changed [15].

### Estimated endemic and threatened species richness

According to our data, larger PAs are more efficient, i.e. have higher species richness, than smaller PAs. Relationship between the size of Amazonian PAs and potential Amazon species richness was positive, but with a low explaining power. The low coefficient of determination obtained (R^2^ = 0.43) was influenced by the indigenous lands Alto Turiaçu, Alto Rio Guama, Awa, and Caru, and the conservation unit REBIO Gurupi, which altogether assemble the forest block of Gurupi, totaling 13,900 km^2^. These distinct PAs, have distinct kinds of usage, and so were analyzed separately, but biologically they seem to be in fact a unit, having a similar species richness as bigger PAs (APA Baixada Maranhense, 17,285 km^2^ and APA Reentrâncias Maranhenses, 26,285 km^2^). Considering the forest block of Gurupi as a unique PA would increase the coefficient of determination, and so the positive relationship (data not shown). This relationship is in agreement with previous findings [112,113]. Peres [112] further states that only a well-connected network of mega-reserves, exceeding an area of 10,000 Km^2^, would cover a major portion of regional biodiversity, preserving populations of rare predators, but also species with seasonal movements (e.g. *G*. *guarouba* and *A*. *xanthops*), and animals impacted by hunting (e.g. among our target taxa, *P*. *obscurus* and *C*. *f*. *pinima*). In fact, within eastern Amazon, roads seem to impact on avian species richness and composition due to habitat fragmentation but also by facilitating logging, fire, hunting, and other traffic disturbances [114]. Additionally, considering the potential effects of the predicted climate changes upon overall biodiversity, patch connectivity may become even more important to guarantee species dispersal in the future. Mega-reserves are considered to enable species to better overcome climatic changes than smaller PAs [115], since larger areas potentially enable species to maintain larger population sizes, with greater genetic diversity, allowing them to adapt their niches and distributions in changing environments [113].

Within Cerrado, we did not find a relationship between PA size and species richness, but Cerrado PAs are mostly misallocated, covering areas of low species richness, and not suitable for cultivation. As abovementioned, Cerrado PAs are failing in protecting the biome´s biodiversity (this study;[39]).

Our results agree with Marini et al. [39], Bini et al. [6], and Peres [112] in that more (and larger) PAs are needed to maintain eastern Amazon and Cerrado biodiversity. Noteworthy, both Amazon and Cerrado PAs face the same anthropogenic pressure as other Neotropical regions [116–118], so similar studies are still needed throughout the Neotropics to review the systems of PAs (but see [119–121]).

### Identification of priority areas for conservation

Considering the entire BAE, less than 17% of its area is currently protected (1.4% conservation units of full protection, 9.77% conservation units of sustainable use, and 6.49% indigenous lands [65]). Currently, the greatest rates of deforestation, within the Amazon, occur precisely in the east, due to a stronger pressure from economic groups that occupy public and private lands for the development of agricultural production, logging and cattle-raising [122]. One of the most important areas currently protected, REBIO Gurupi, has also lost 20% of its area due to illegal occupation for agricultural exploitation, extraction of wood, burning and deforestation made by squatters and other landowners [67,123–125]. REBIO Gurupi is part of the forest block of Gurupi, that together with APA Baixada Maranhense, and APA Reentrâncias Maranhenses were identified as Important Bird Areas (IBAs), with the occurrence of Endangered and Near Threatened species’ populations and “trigger species”, and also considered of "extreme importance" (this study;[71,103,124]). Thus, and according to Peres’ [112] recommendations to extend PAs networks into mega-reserves, we highlight the regions connecting APA Baixada Maranhense with indigenous land Alto Turiaçu, and an extension of this last PA as priority for conservation actions.

Cerrado holds 5% of the planet's biodiversity and is considered the richest savanna in the world, but one of the most threatened regions in Brazil, which has lost about 48% of its native vegetation until 2008, and has only 2.2% of protected area [60,62,69]. Estimates indicate that at least 20% of endemic and threatened species within the whole biome remain outside parks and reserves [126]. As extensively debated above, Cerrado system of PAs needs to be revised (this study, [6,39]), but the better location of new PAs has been contentious. According to Bini et al. [6], weighting for the knowledge on species distribution, new areas in the north of the biome should be priority, but for Marini et al. [39] and Diniz-Filho et al. [127,128], these new areas should be in the southern part of the biome, since species richness was higher there. The priority areas for conservation we suggest, not only had higher species richness and still hold extensive native vegetation, but also were already recommended to acquire conservation unit status by MMA [71] (Polígono das Águas in southern Maranhão, Southwestern Plateau, and Mirador/ Uruçuí). The last area was also indicated as an IBA for the presence of endemic species as *A*. *xanthops*, *M*. *torquata*, *C*. *cristatellus*, *P*. *caerulescens*, *C*. *eucosma*, *S*. *atricollis* and the threatened *C*. *obrieni* [124]. In central Maranhão, we recommend the extension of the Maranhão semideciduous forest area (also already highlighted by [71]).

Finally, our results highlight the importance of indigenous lands in the conservation of Neotropical biodiversity. Among the areas with higher species richness (≥50% of taxa), more than a half were indigenous lands. Brazil's forestry code (Law 12651, Article 3, 25 May 2012) classifies indigenous lands as areas of full protection. However, Rylands [129] and Instituto Socioambiental et al. [130] categorize indigenous lands as areas that allow human occupation and/or sustainable management activities, having a conflicting view about the land uses that should be allowed in these areas [131]. Given the value of indigenous lands for conservation, the development of community management plans is essential to conserve the biological resources of the region, and is beneficial for all society [67].

## Conclusions

Protected Areas in Eastern Amazon are large and, at least in part, well connected, holding high biodiversity. Nonetheless, the lack of overall biological knowledge, and the high rate of deforestation, habitat degradation, and mostly economic pressures make studies such as ours only useful if accompanied by an increase of public awareness, adequate governmental policy, and proper conservation planning. Noteworthy, this is most striking in Cerrado, where scientific debate on conservation actions has been quite intense and controversial, but habitat degradation has increased. Nonetheless, our results further validate governmental reports on the implementation of new PAs, and encourage putting these findings into practice.

## Supporting information

S1 Fig Occurrences for the taxa analyzed. A-X) Twenty-four maps depicting 929 records obtained from the literature (triangles), museum collections (diamonds), online databases (crosses), and field expeditions (circles).
(PDF)

S2 Fig Species distribution models. First map depicts the extent area in yellow, and the study area in red, Amazon 1) and Cerrado 2) biomes, and all the other maps represent the 24 SDMs estimated. SDMs for *Herpsilochmus longirostris* and *Alipiopsitta xanthops* include potential distributions overlaid by the border of the study area.
(TIF)
