# Supplementary material for: Delimiting priority areas for the conservation of endemic and threatened Neotropical birds using a niche-based gap analysis
Source: PLoS One. 2017 Feb 10;12(2):e0171838. doi: 10.1371/journal.pone.0171838 (PMC5302823; doi:10.1371/journal.pone.0171838)

S1 Occurrences for the taxa analyzed. A-X) Twenty-four maps depicting 929 records obtained from the literature (triangles), museum collections (diamonds), online databases(crosses), and field expeditions (circles).

(A) Crax fasciolata pinima

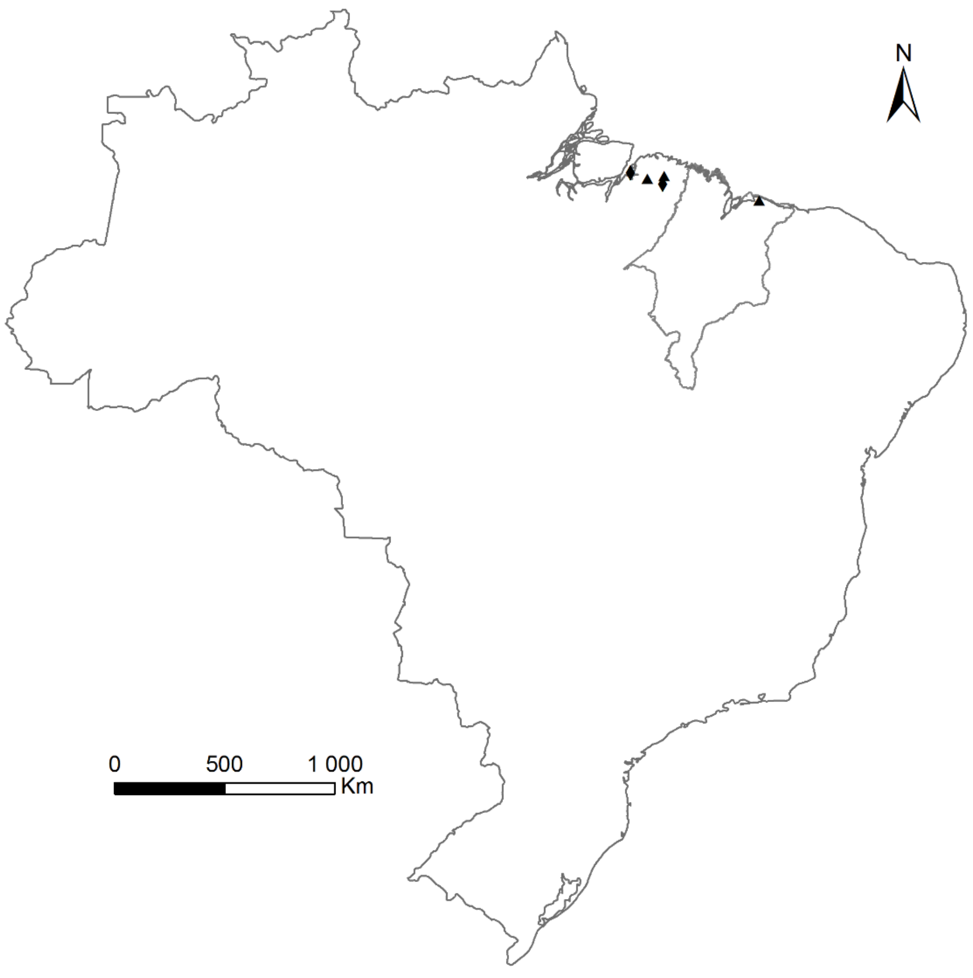

(B) Psophia obscura

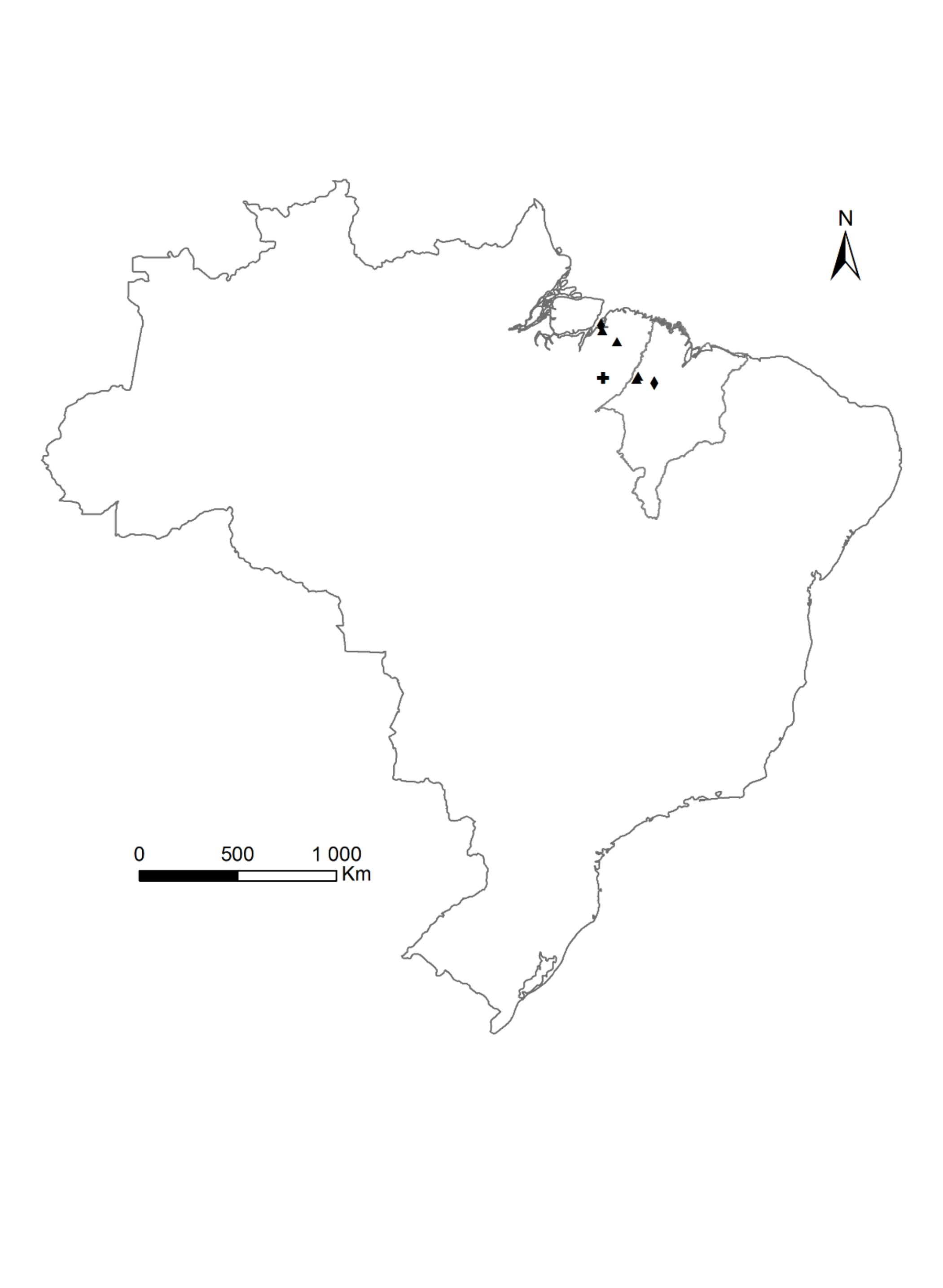

(C) Guaruba guarouba

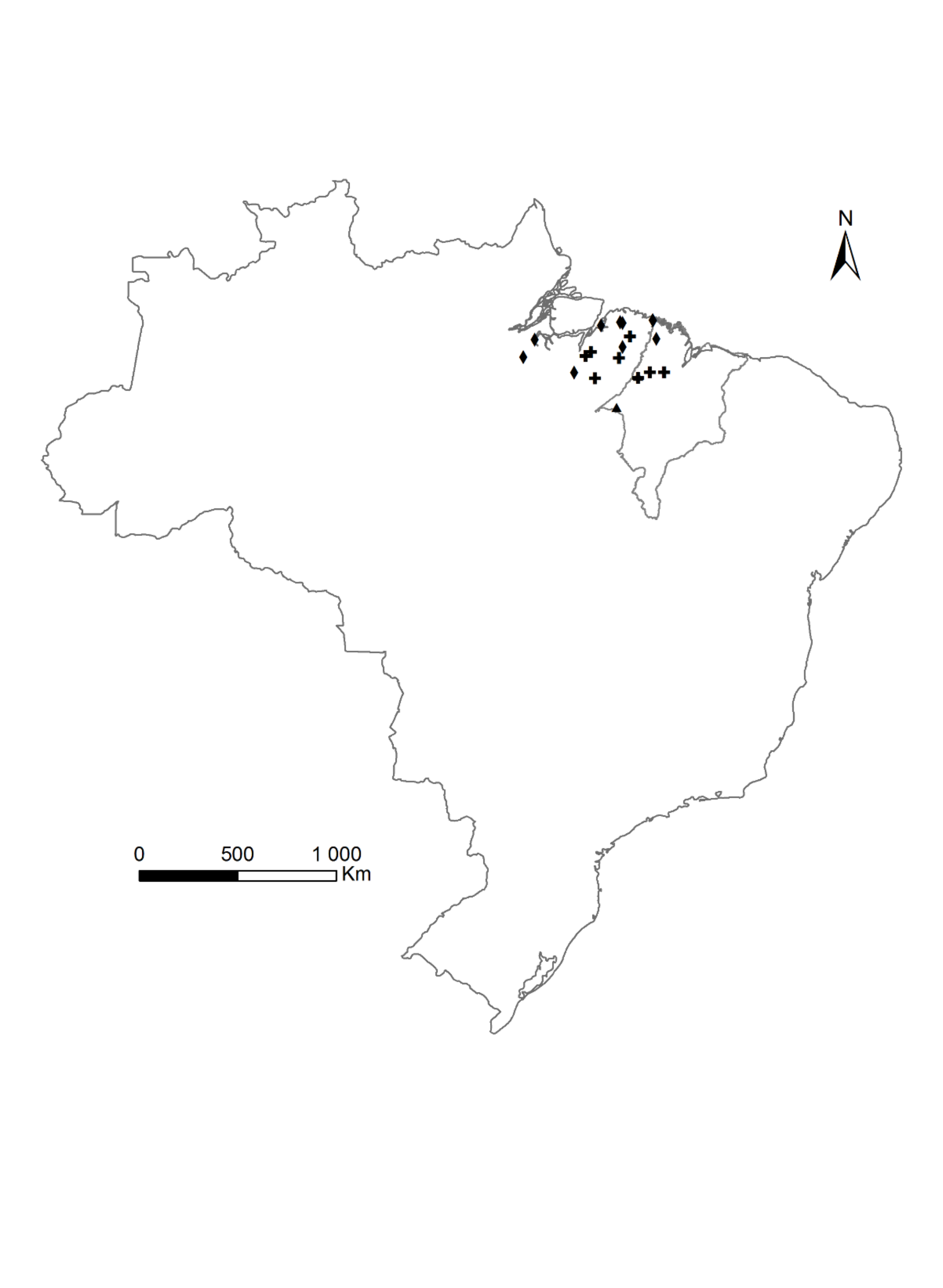

(D) Pyrrhura coerulescens

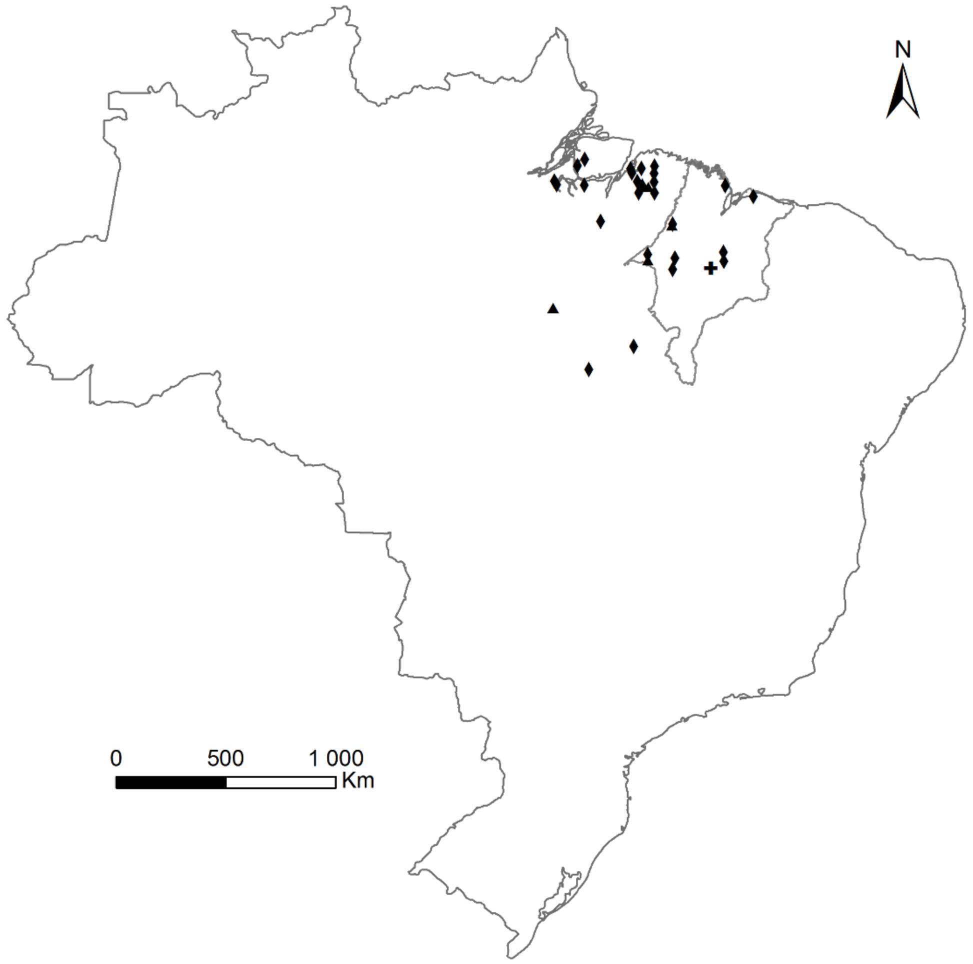

(E) Neomorphus geoffroyi

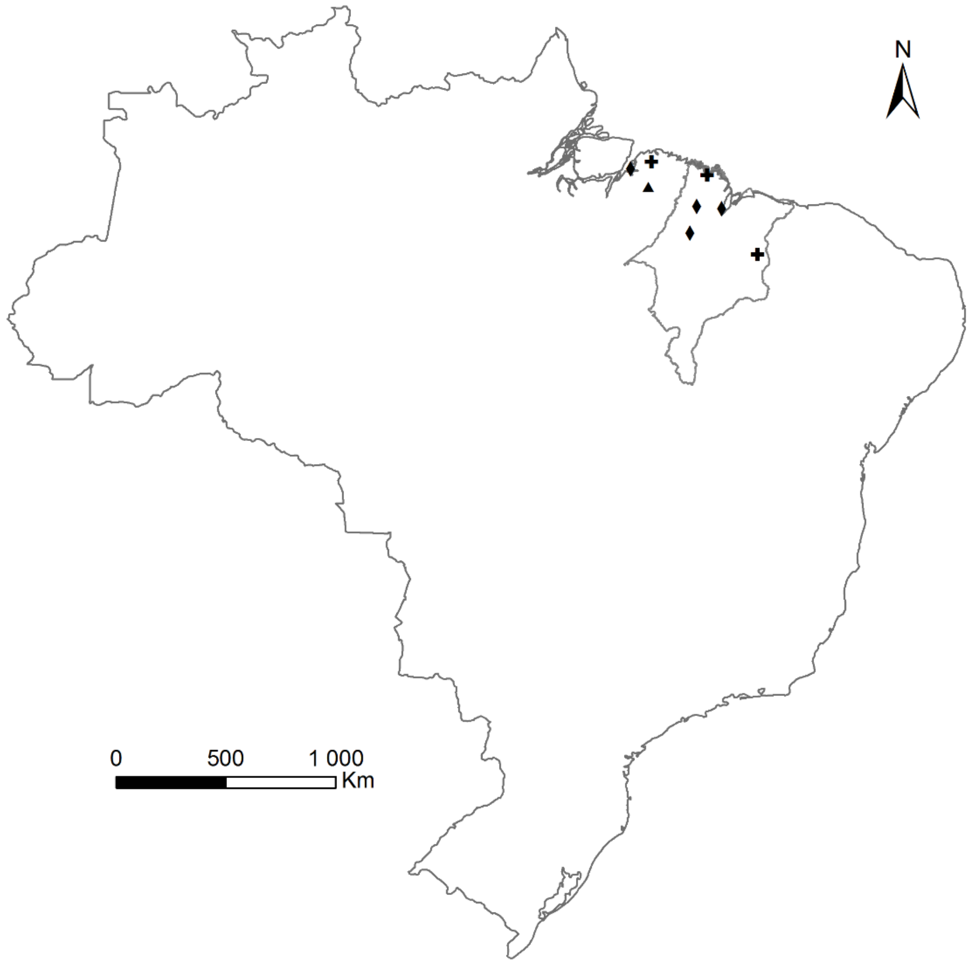

(F) Pteroglossus bitorquatus bitorquatus

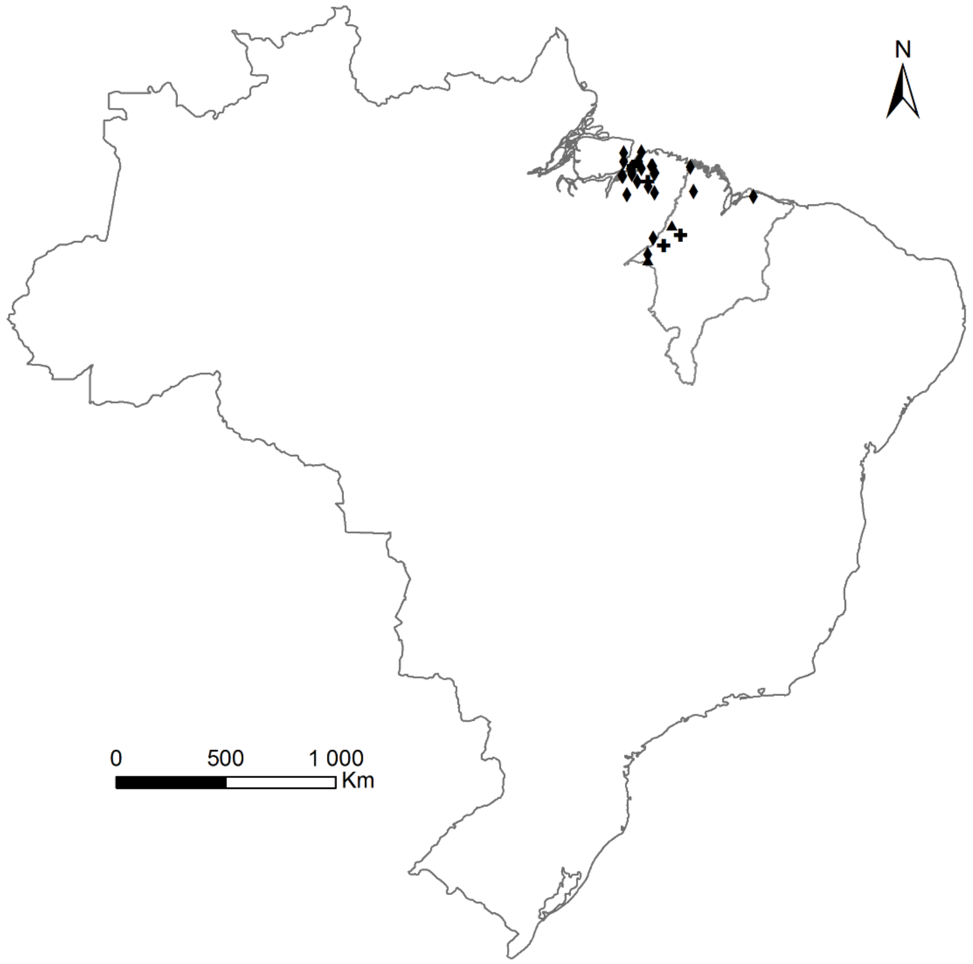

(G) Celeus obrieni

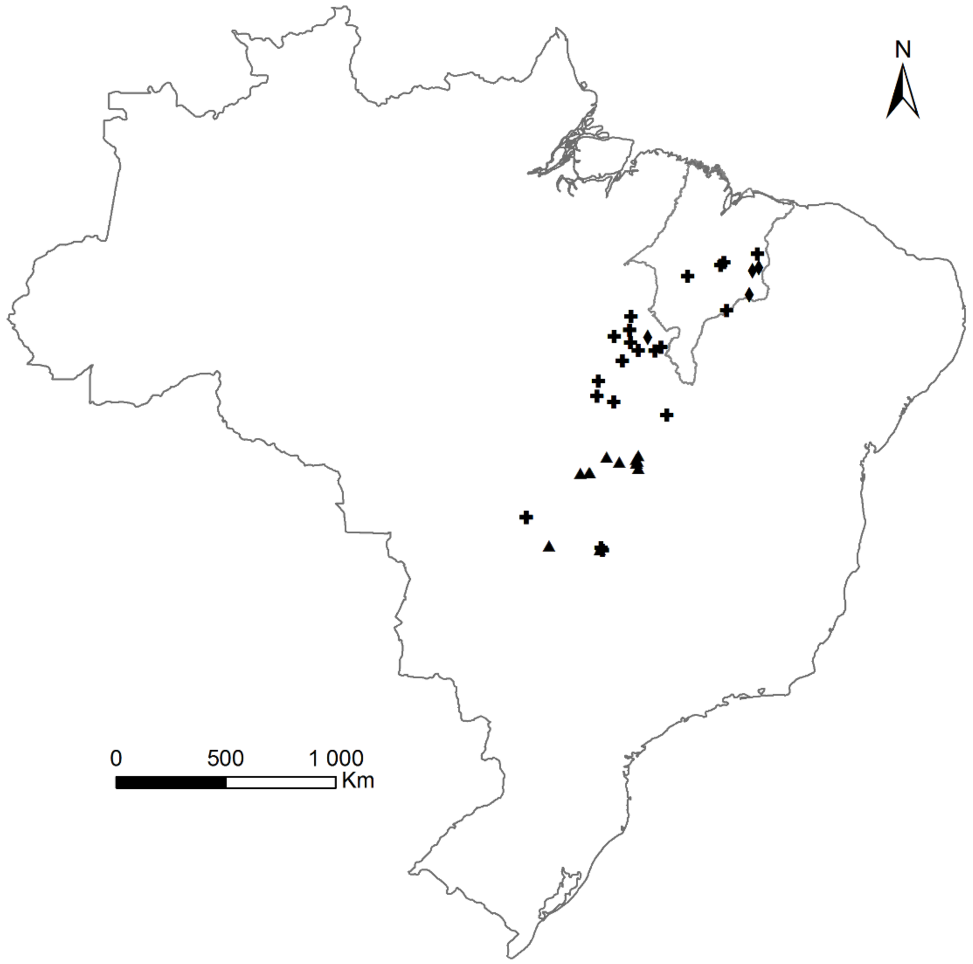

(H) Piculus paraensis

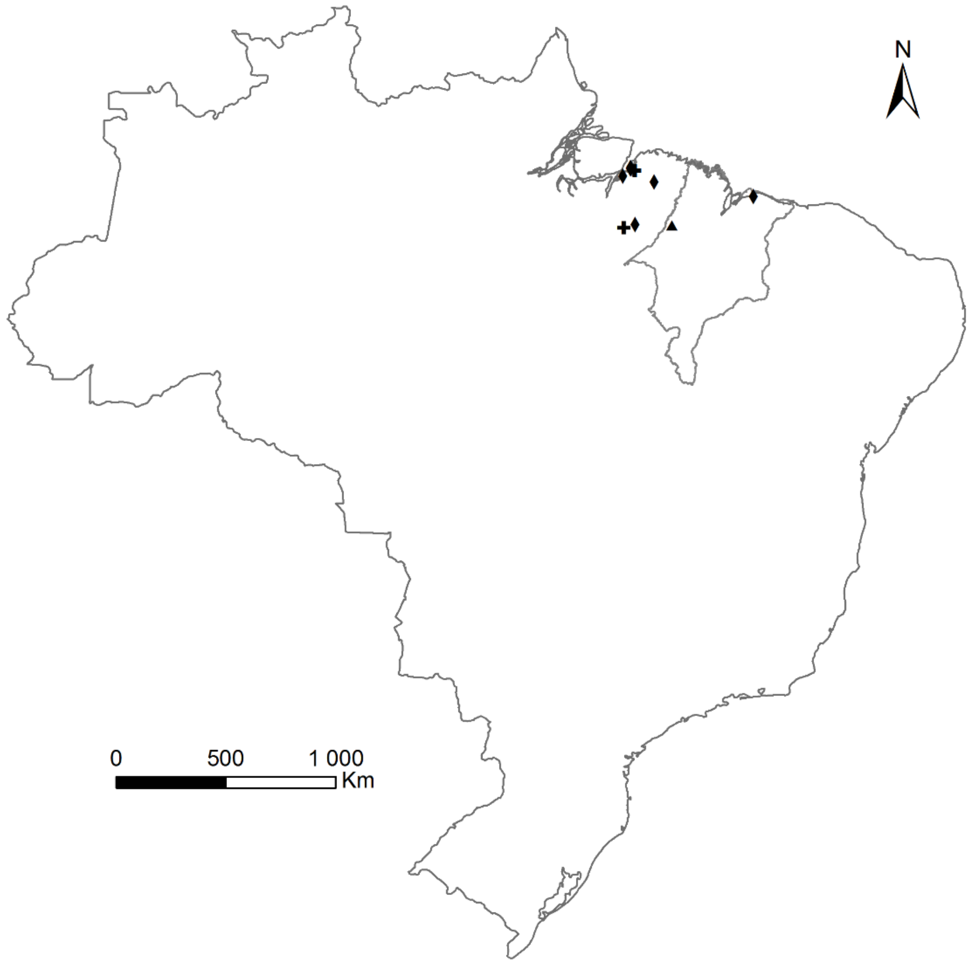

(I) Phlegopsis nigromaculata paraensis

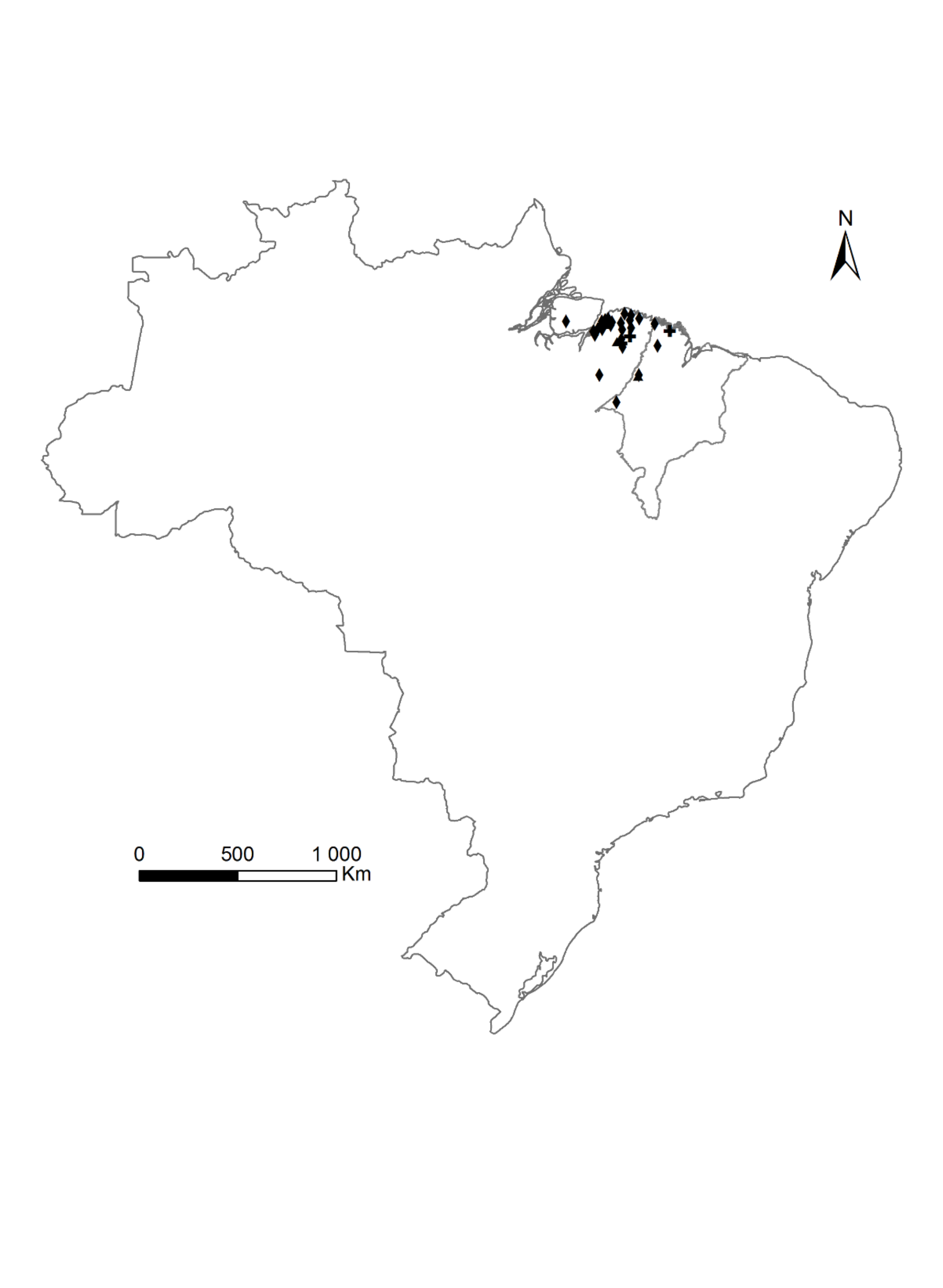

(J) Hylopezus paraensis

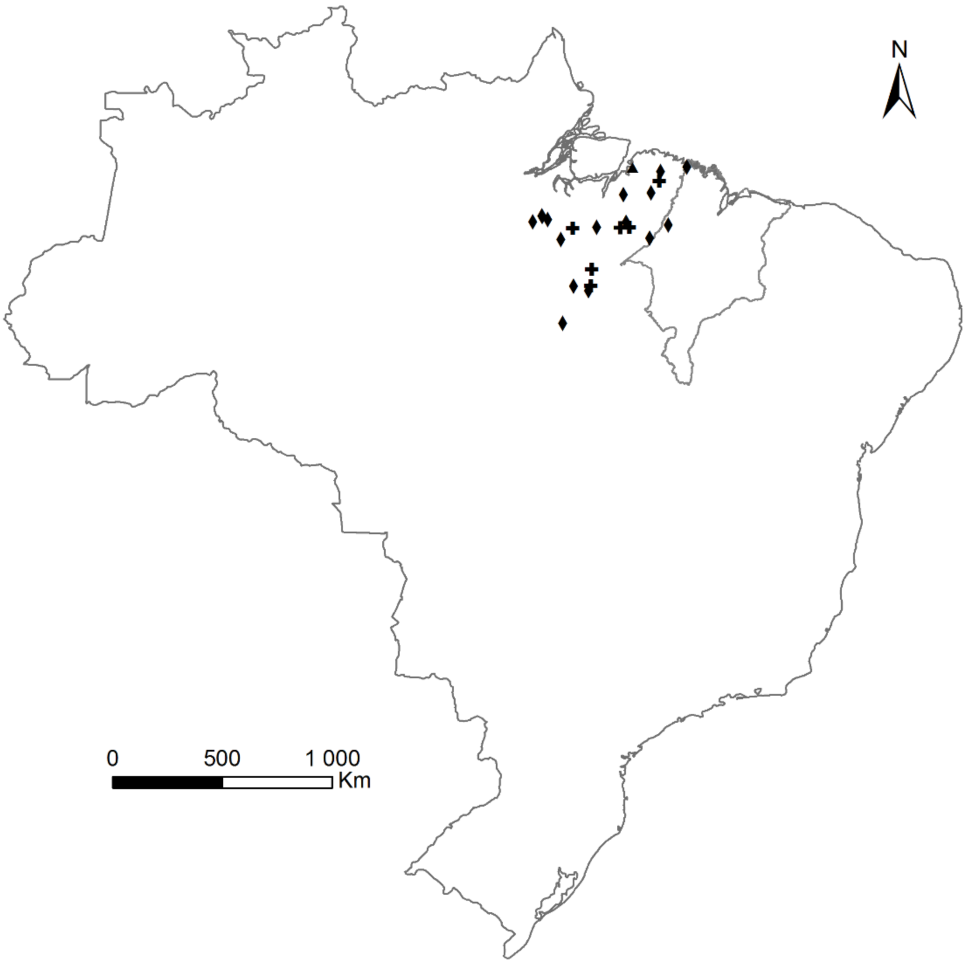

(K) Dendrocincla merula badia

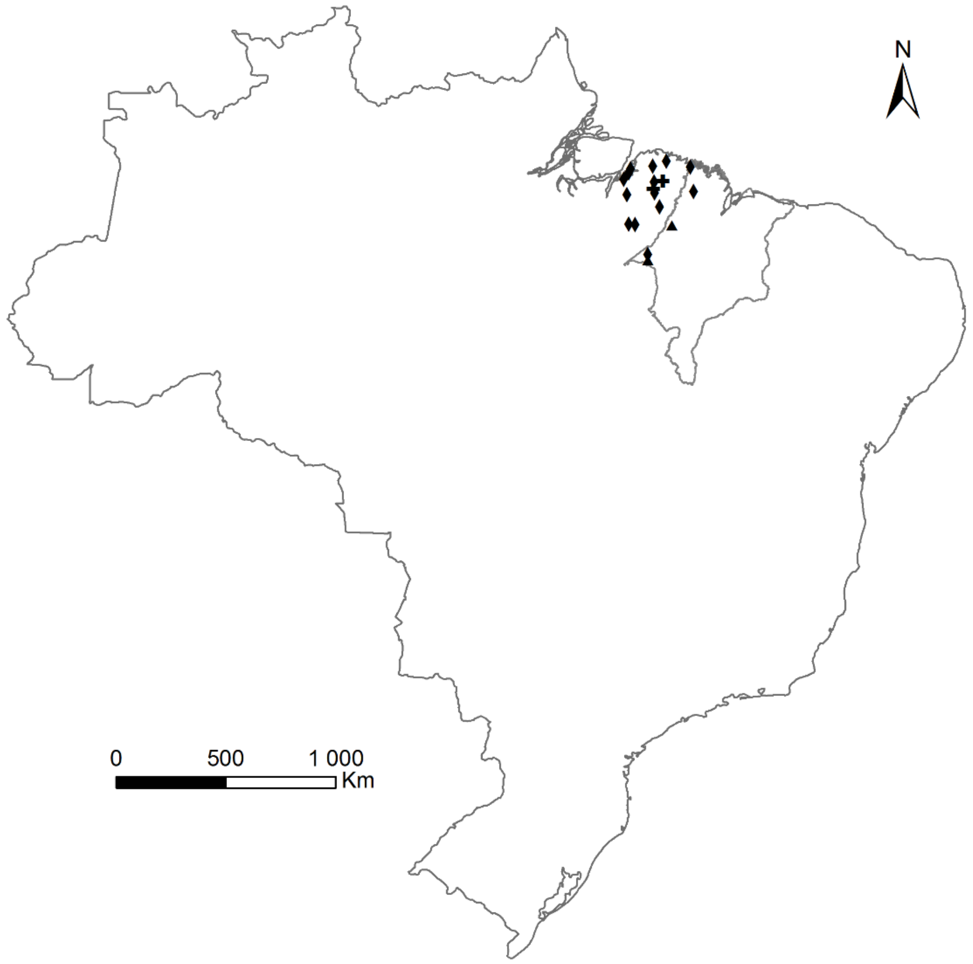

(L) Dendrexetastes rufigula paraensis

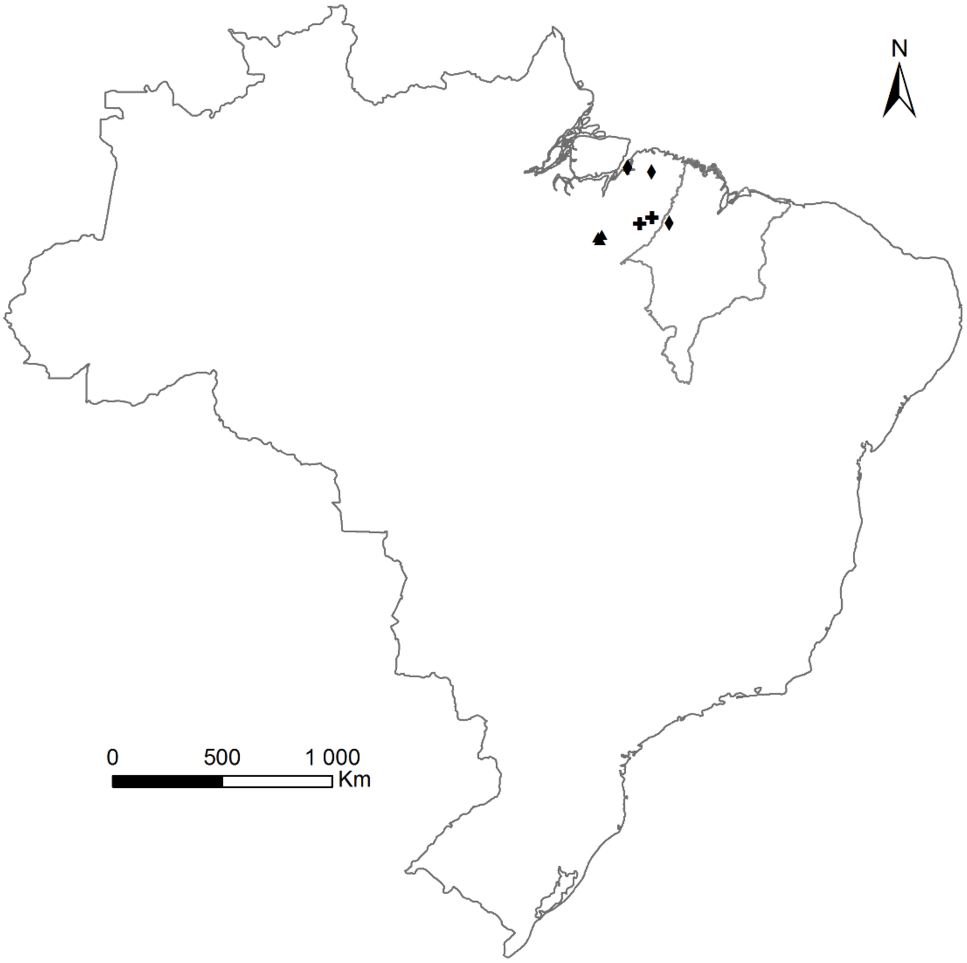

(M) *Dendrocolaptes medius*

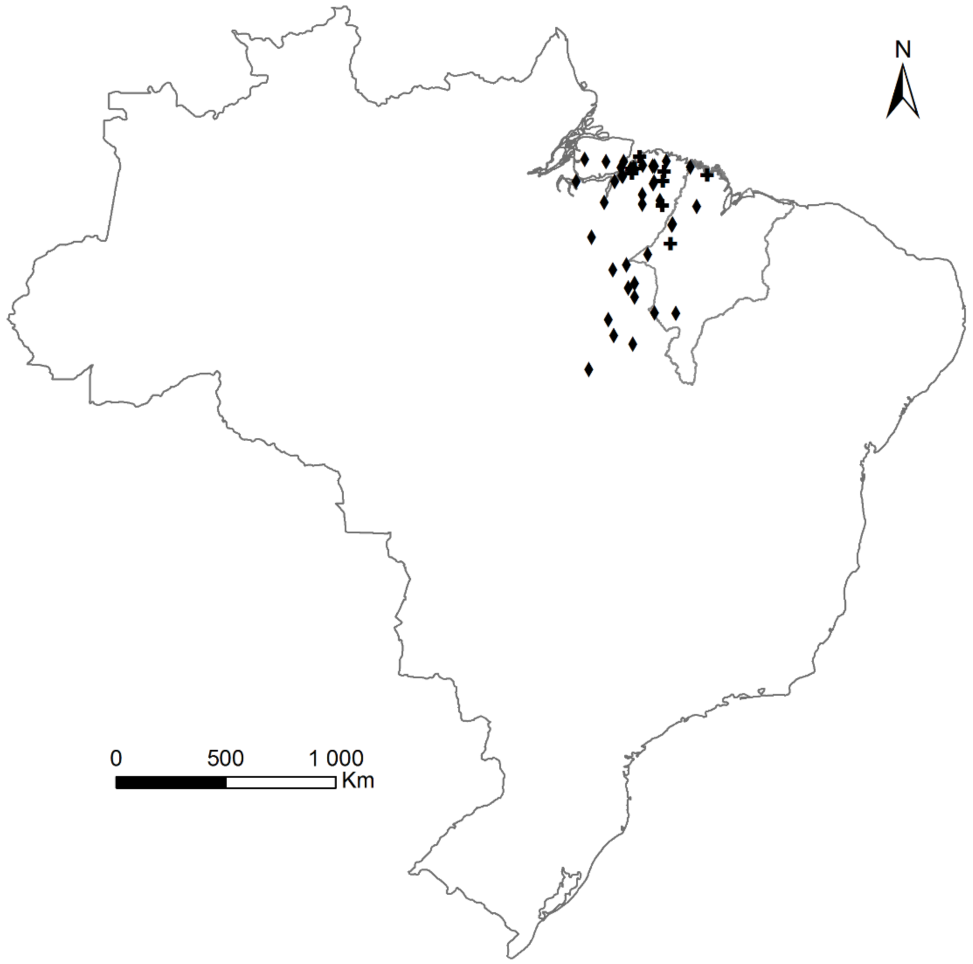

(N) Hylophilus ochraceiceps rubrifrons

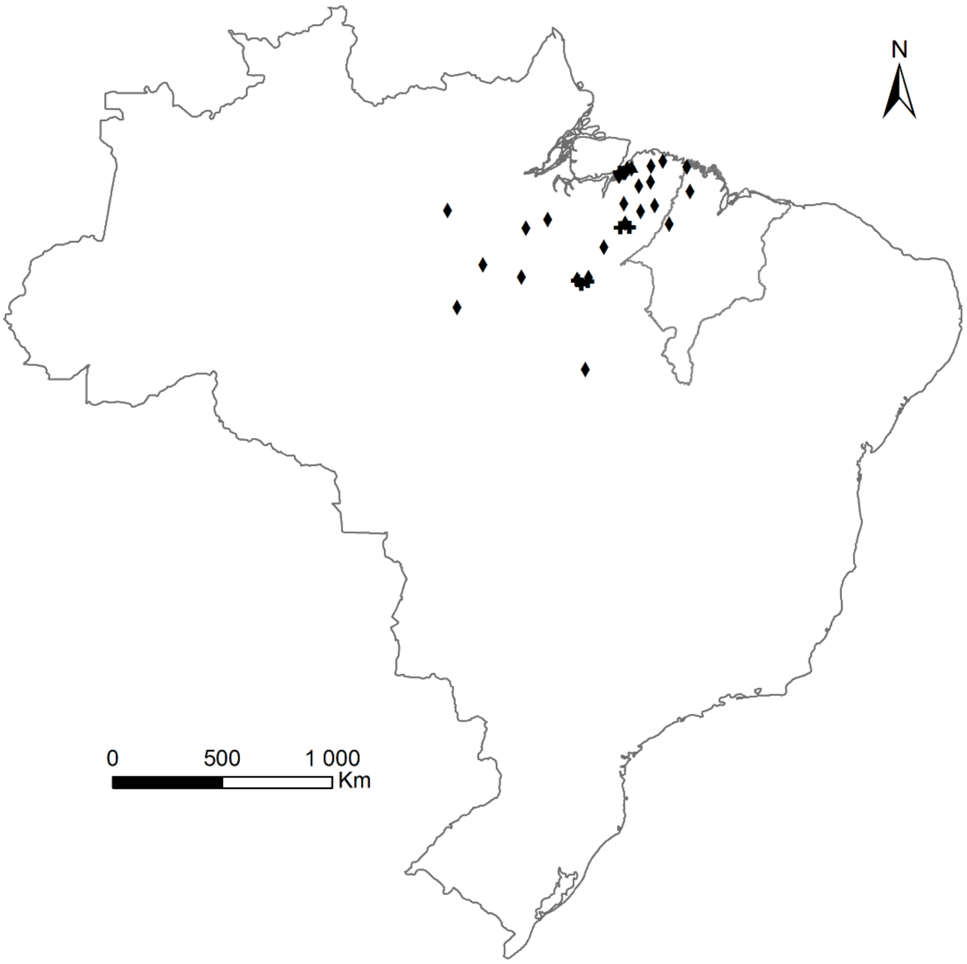

(O) Alipiopsitta xanthops

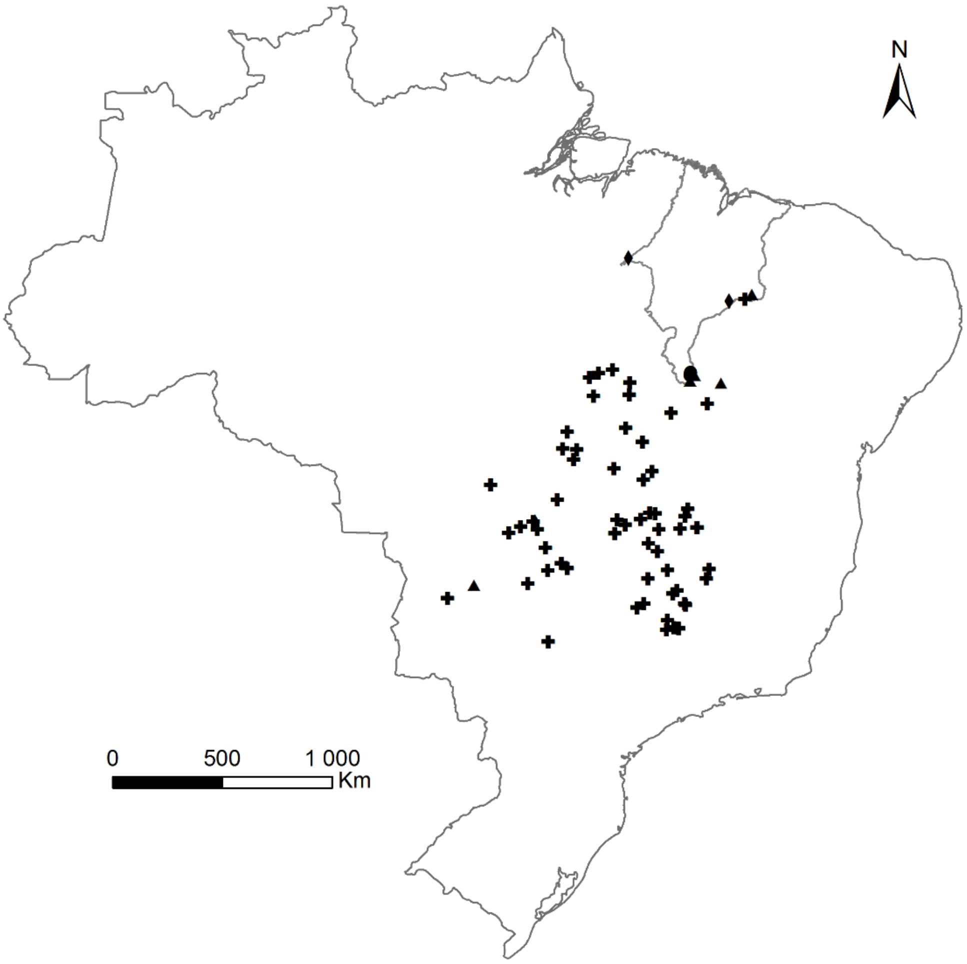

(P) Cercomacra ferdinandi

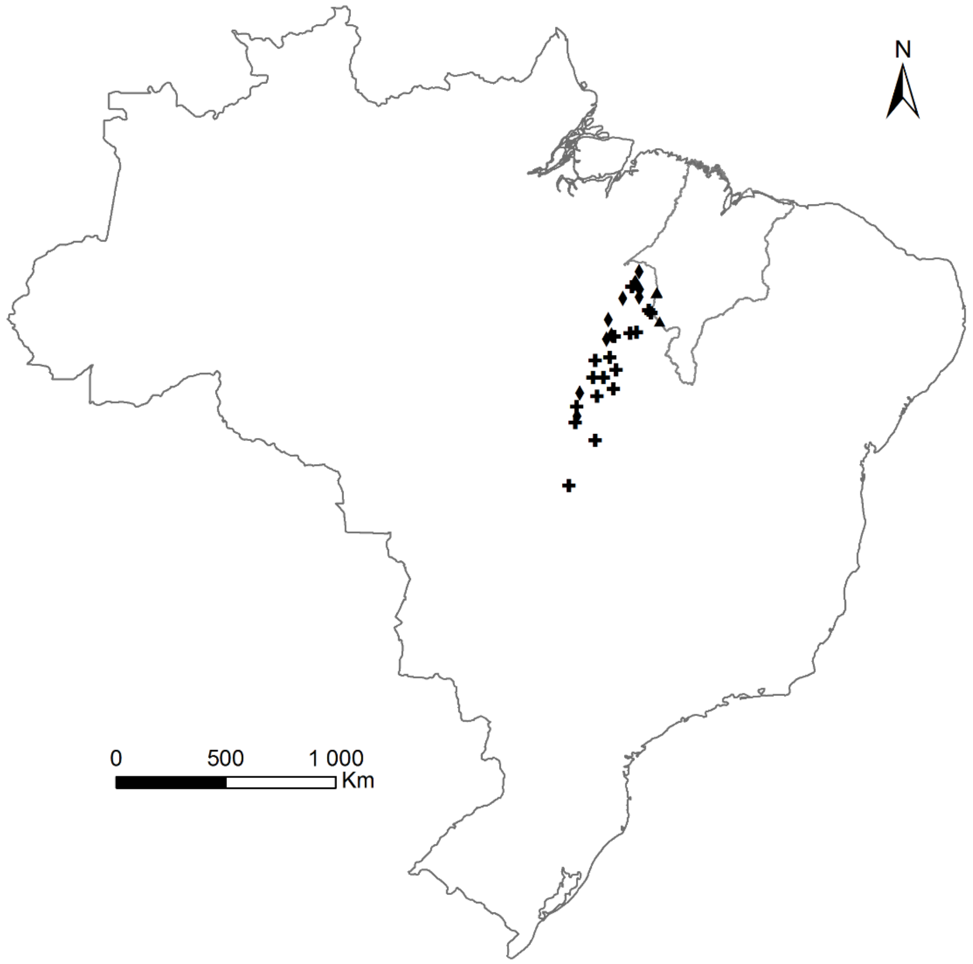

(Q) Herpsilochmus longirostris

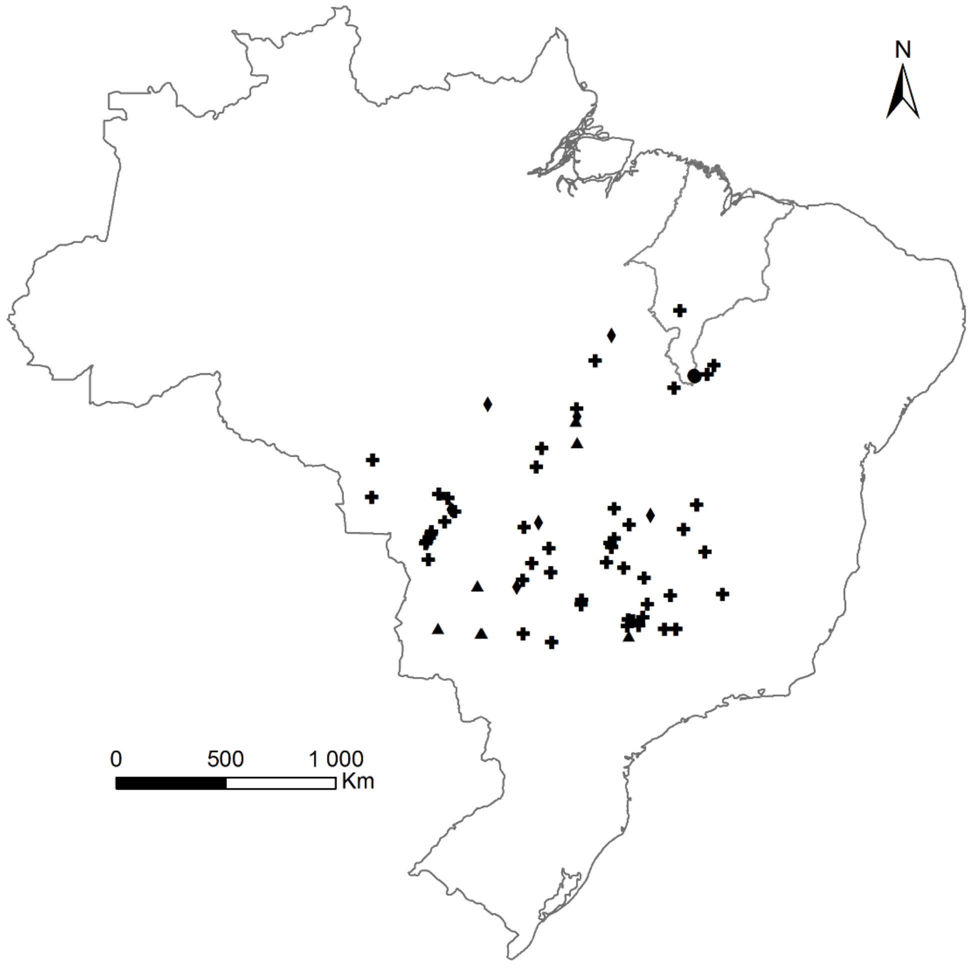

(R) Melanopareia torquata

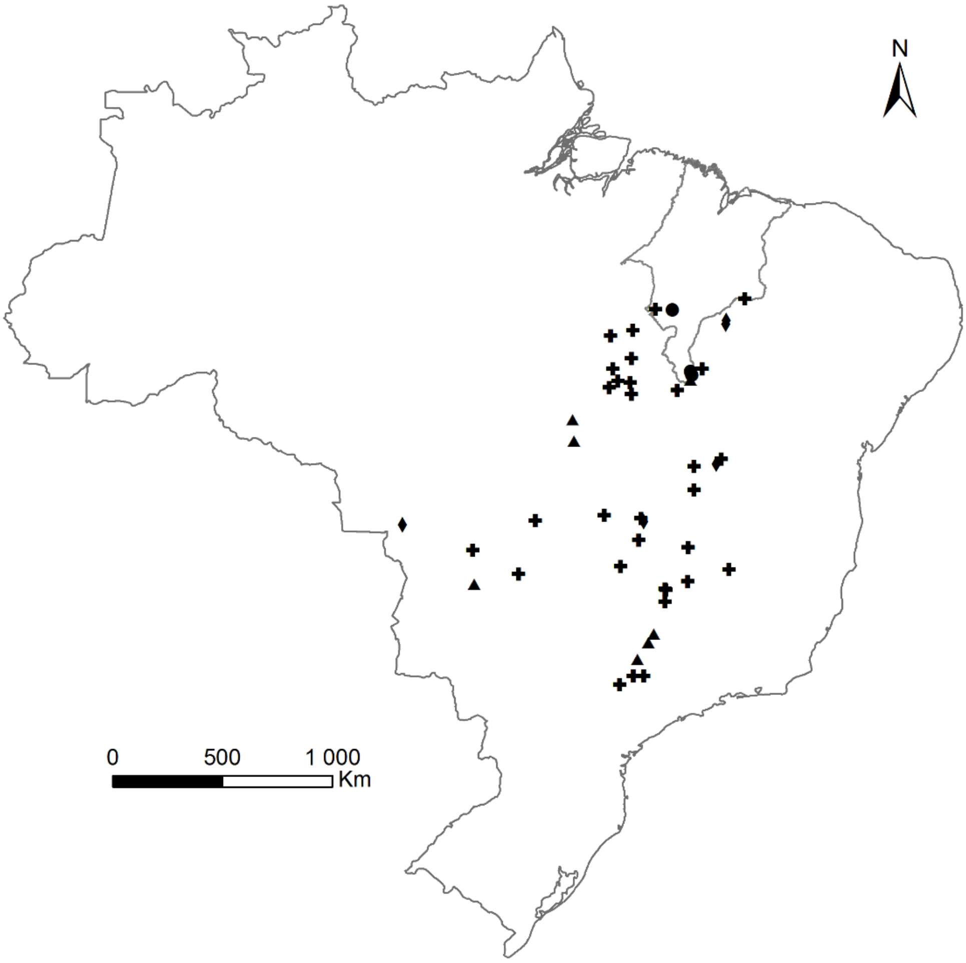

(S) Antilophia galeata

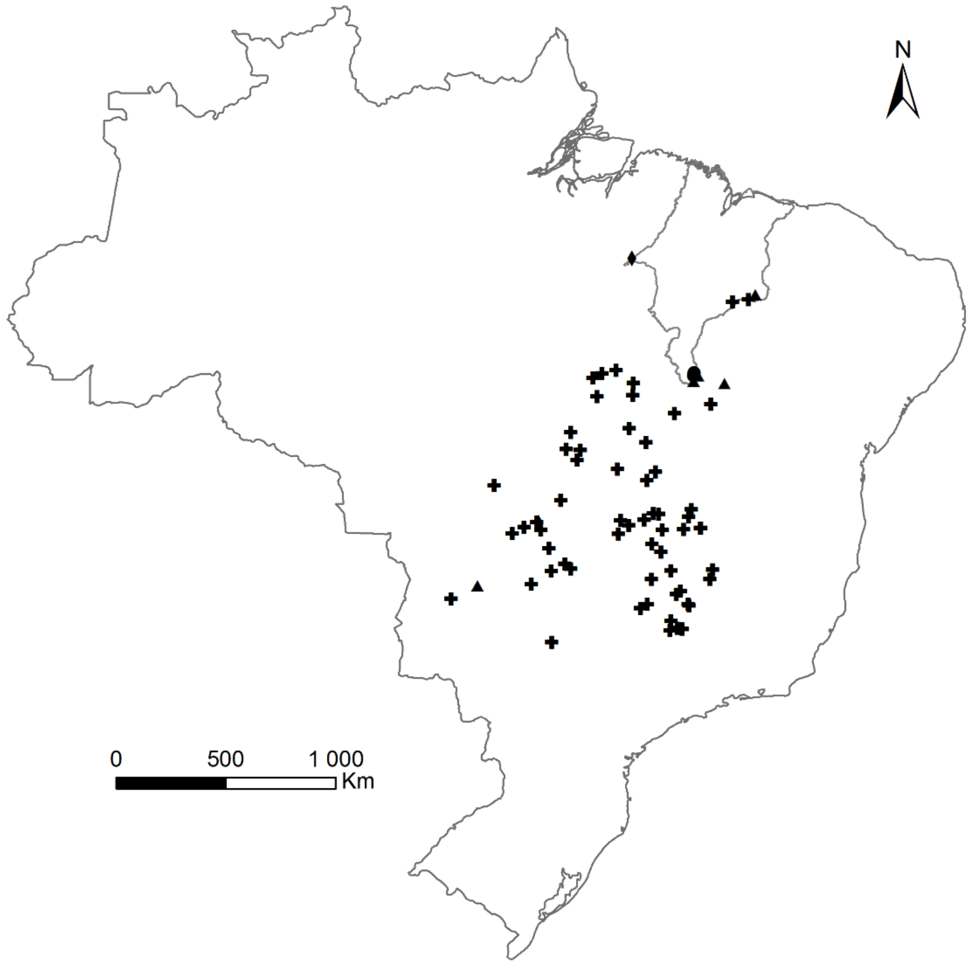

(T) *Suiriri affinis*

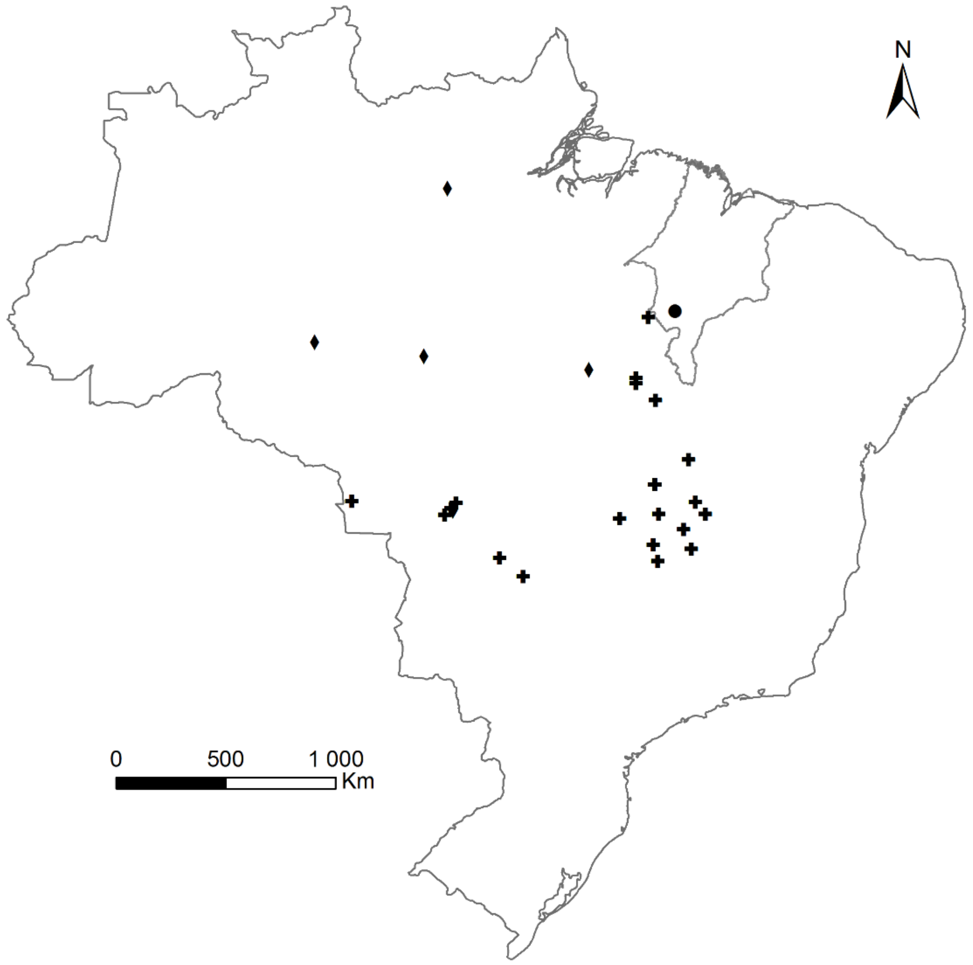

(U) Cyanocorax cristatellus

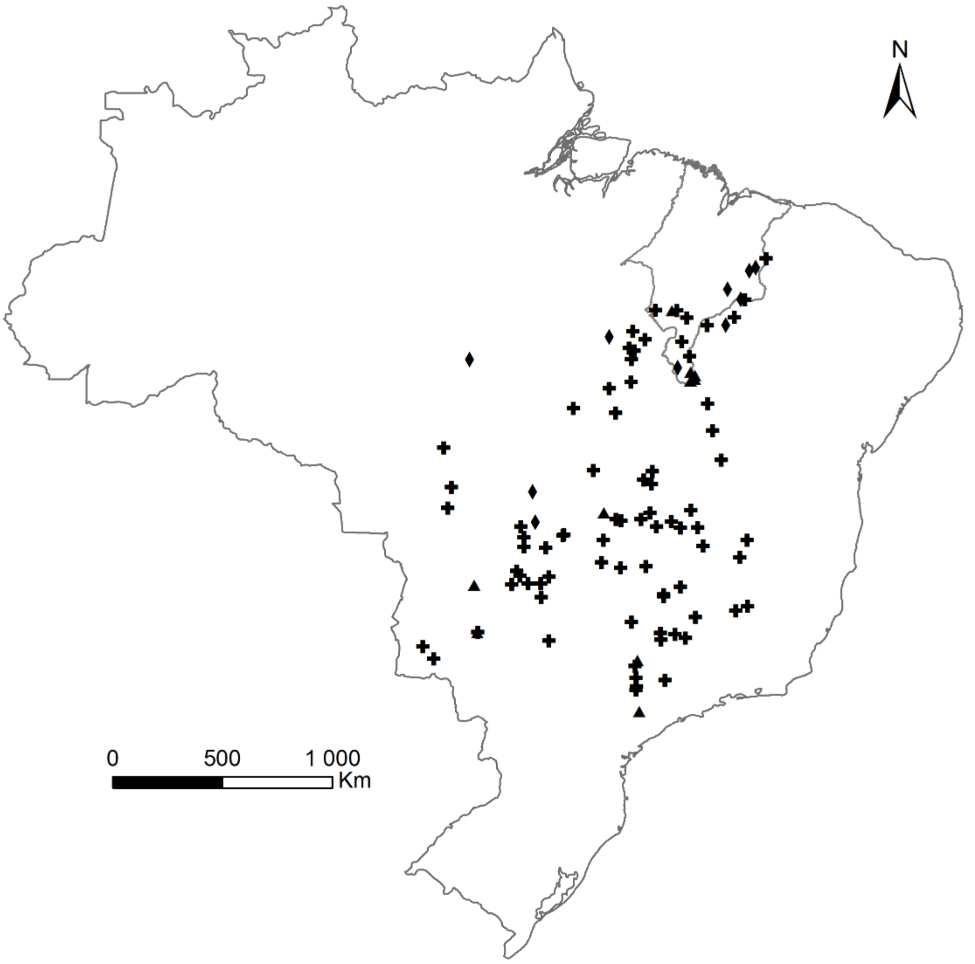

(V) Charitospiza eucosma

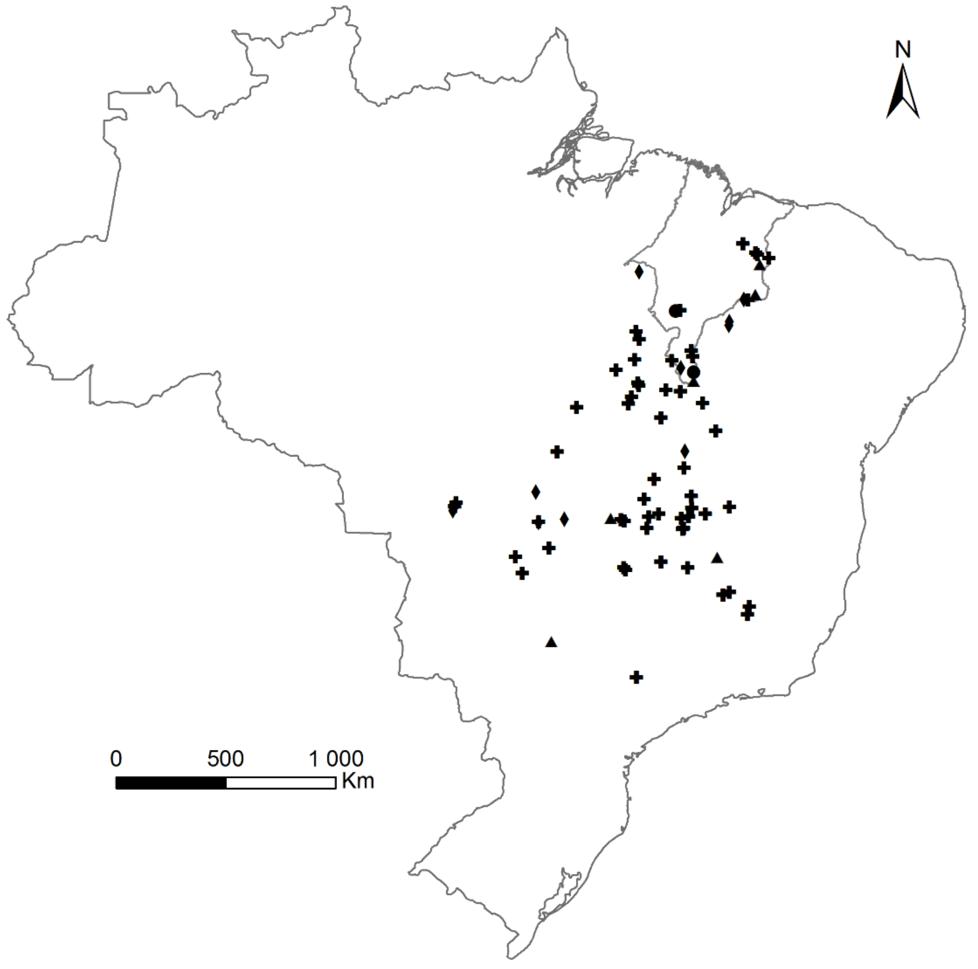

(W) Saltatricola atricollis

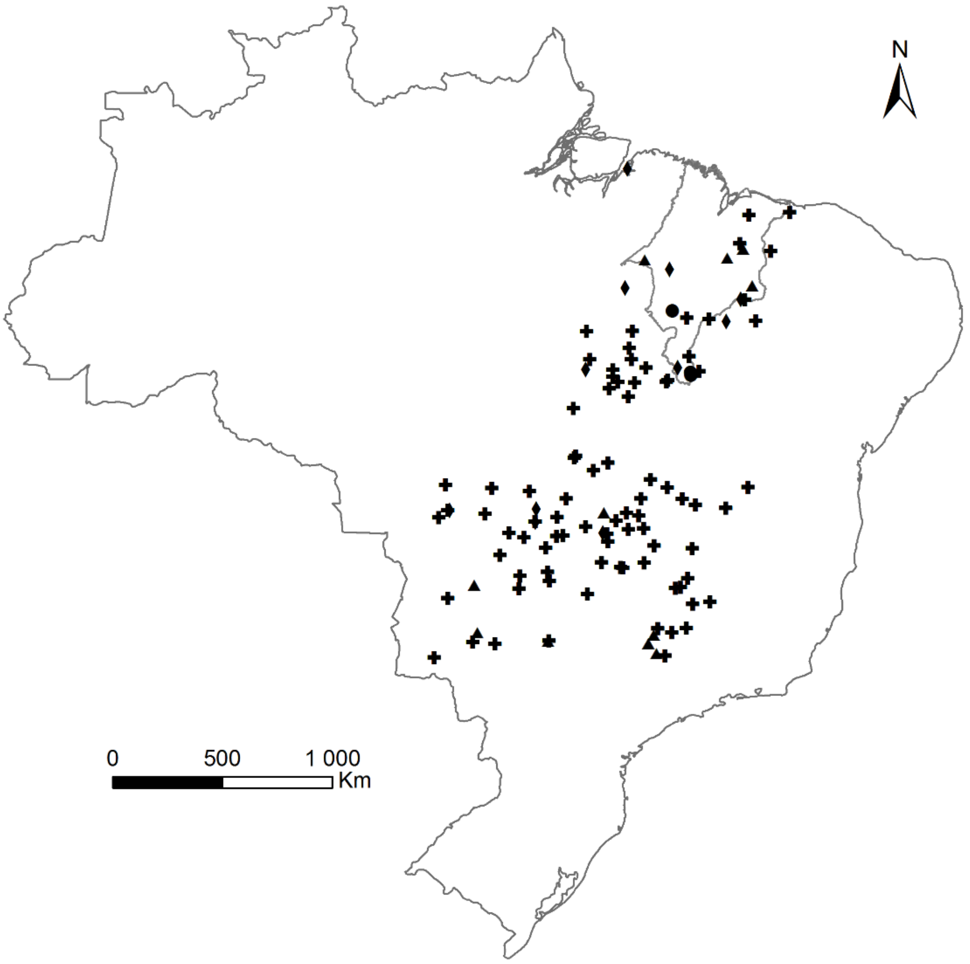

(X) Porphyrospiza caerulescens

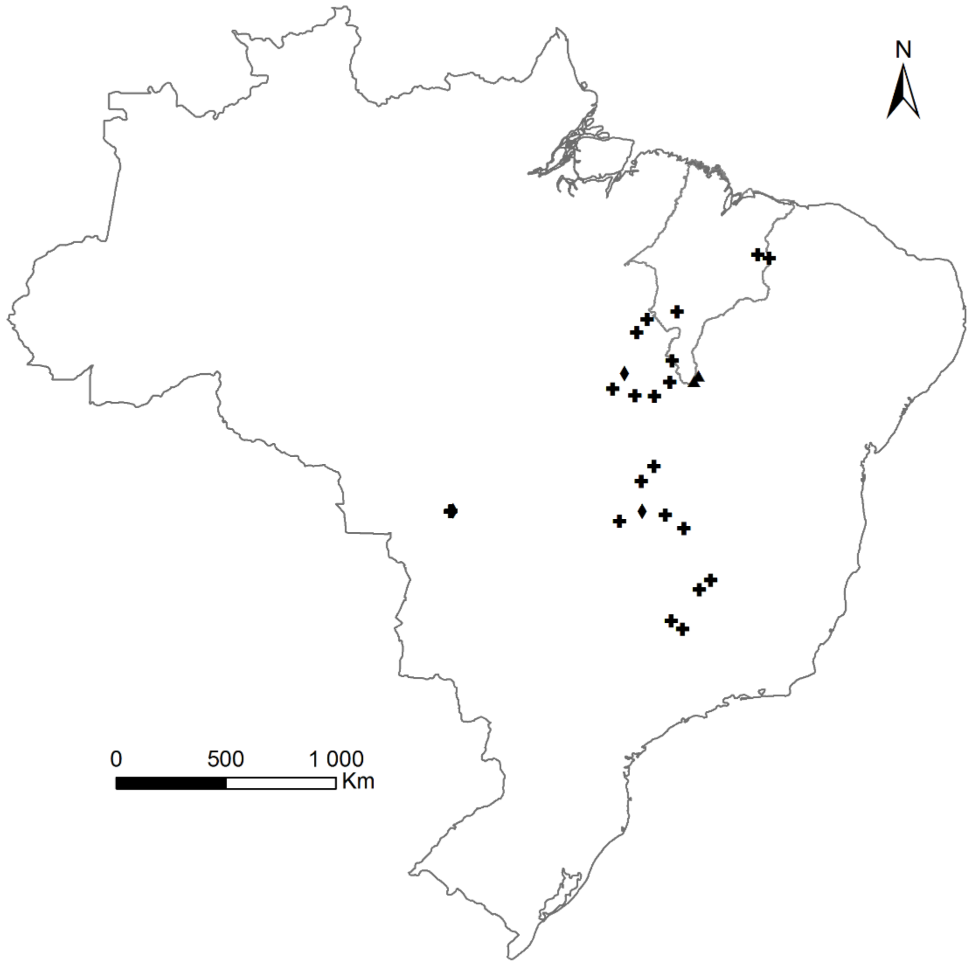

Supplement: S1 Fig — A-X) Twenty-four maps depicting 929 records obtained from the literature (triangles), museum collections (diamonds), online databases (crosses), and field expeditions (circles). (PDF) [file pone.0171838.s001.pdf]
