# Supplementary figures and images for: Delimiting priority areas for the conservation of endemic and threatened Neotropical birds using a niche-based gap analysis
Source: PLoS One. 2017 Feb 10;12(2):e0171838. doi: 10.1371/journal.pone.0171838 (PMC5302823; doi:10.1371/journal.pone.0171838)

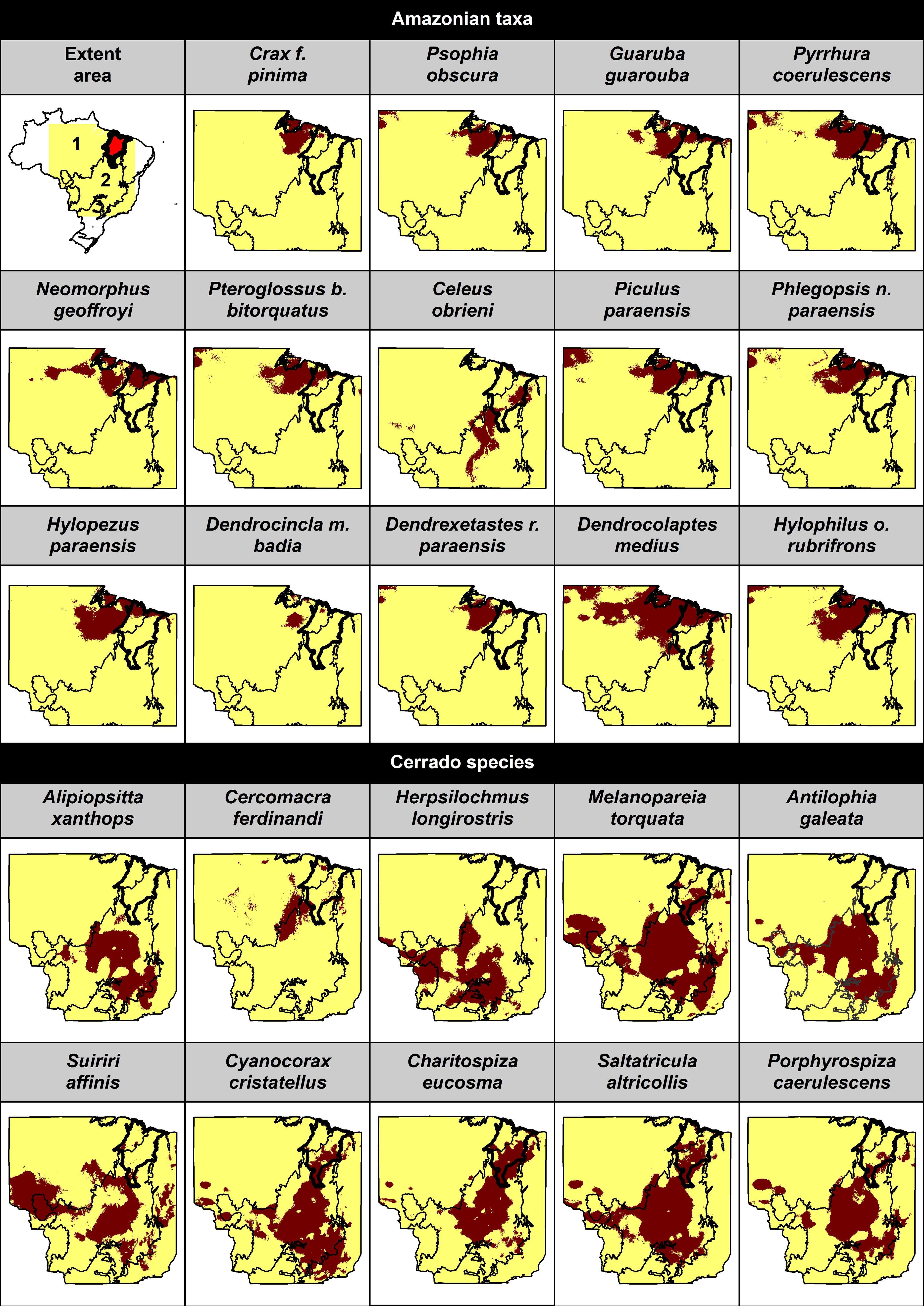

Supplement: S2 Fig — First map depicts the extent area in yellow, and the study area in red, Amazon 1) and Cerrado 2) biomes, and all the other maps represent the 24 SDMs estimated. SDMs for Herpsilochmus longirostris and Alipiopsitta xanthops include potential distributions overlaid by the border of the study area. (TIF) [file pone.0171838.s002.tif]
